# Retinal microvascular changes in diabetic patients with diabetic nephropathy

**DOI:** 10.1186/s12902-022-01250-w

**Published:** 2023-05-05

**Authors:** Yujie Yan, Liping Yu, Chuan Sun, Haipeng Zhao, Hongsong Zhang, Zhijun Wang

**Affiliations:** 1 Department of Ophthalmology, China-Japan, Friendship Hospital, Beijing, People’s Republic of China; 2 Department of Endocrinology, China-Japan, Friendship Hospital, Beijing, People’s Republic of China

**Keywords:** Type 2 diabetes mellitus, Diabetic retinopathy, Diabetic nephropathy, Hard exudates, Urine albumin creatine ratio

## Abstract

**Background:**

To explore the characteristics of retina microvascular changes in patients with diabetic nephropathy (DN) and its risk factors.

**Methods:**

Retrospective, observational study. 145 patients with type 2 diabetic mellitus (DM) and DN were included in the study. Demographic and clinical parameters were obtained from medical records. Presence of diabetic retinopathy (DR), hard exudates (HEs) and diabetic macular edema (DME) were evaluated according to the color fundus images, optical coherence tomography (OCT) and fluorescence angiography (FFA).

**Results:**

DR accounted for 61.4% in type 2 DM patients with DN, of which proliferative diabetic retinopathy (PDR) accounted for 23.6% and sight threatening DR accounted for 35.7%. DR group had significantly higher levels of low-density lipoprotein cholesterol (LDL-C) (*p* = 0.004), HbA1c (*P* = 0.037), Urine albumin creatine ratio (ACR) (*p* < 0.001) and lower level of estimated glomerular filtration rate (eGFR) (*P* = 0.013). Logistic regression analysis showed DR was significantly associated with ACR stage (*p* = 0.011). Subjects with ACR stage3 had higher incidence of DR compared with subjects with ACR stage1 (OR = 24.15, 95%CI: 2.06–282.95). 138 eyes of 138 patients were analyzed for HEs and DME, of which 23.2% had HEs in posterior pole and 9.4% had DME. Visual acuity was worse in HEs group than in non-HEs group. There was significant difference in the LDL-C cholesterol level, total cholesterol (CHOL) level and ACR between HEs group and non-HEs group.

**Conclusions:**

A relatively higher prevalence of DR was found in type 2 DM patients with DN. ACR stage could be recognized as a risk factor for DR in DN patients. Patients with DN needs ophthalmic examination more timely and more frequently.

**Supplementary Information:**

The online version contains supplementary material available at 10.1186/s12902-022-01250-w.

## Background

Diabetes mellitus (DM) is a chronic hyperglycemic state characterized by defects in insulin secretion or/and insulin action [1] and has become a major public health challenge worldwide. The prevalence of diabetes has increased by about tenfold during the past three decades in China [2]. As the prevalence of DM increases, the prevalence of its complications is expected to rise as well, which will be significant burdens for these individuals and on our health care systems.

Diabetic retinopathy (DR) is one of the most common microvascular complications of DM, which is the leading cause of preventable blindness in working-age people. The prevalence of DR in the general population and diabetic patients in a meta-analysis was 1.7% and 22.4% respectively. The highest prevalence of DR was found in patients aged 50–59 years in China [3].

The retina complications of DM result from damage to small vessels, while the kidney complications share the same mechanism. As all belongs to microvascular complications of DM, several investigations have demonstrated the relationship between DR and diabetic nephropathy (DN) in DM patients [4]. Previous studies indicated that DR was significantly associated with renal function deterioration and patients with DN experienced higher incidence of DR as compared with patients without DN [5–7]. Considering that all diabetic microvascular complications share similar etiological factors, screening for DR must be taken in patients with DN [8]

Hard exudates (HEs) are the result of extravasation of fluid, lipids and lipoproteins into the retinal layers from microaneurysms and dilated capillaries [9], which can be frequently observed along with macular edema in DR patients [10]. Several reports have shown that HEs could impact visual abilities. Increasing amounts of exudate appear to be independently associated with an increased risk of visual impairment [11]. Presence of HEs is considered as a sign of recalcitrant macular edema with a poor prognosis for visual recovery [12]. It is reported that diabetic macular HEs disappeared after hemolysis for deteriorating chronic renal failure caused by DN [13], which indicated that renal function and retinal leakage of protein might be paralleled. Thus, relationship between HEs and DN should also be paid attention to when concerning about microvascular complications of DM.

Therefore, we hypothesized that DN severity may be a potential risk factor to microvascular changes in retina. The purpose of our study is to test the hypothesis and to explore the characteristics of retina microvascular changes in patients with DN. These analyses might provide useful information for the prevention and management of DM complications.

## Methods

This was a retrospective, observational, single-center study. The medical records of 145 patients diagnosed with type 2 DM and DN in China-Japan Friendship Hospital from January 2019 to December 2019 were retrospectively reviewed (Additional file 1). This study was approved by the Institutional Review Board of China-Japan Friendship Hospital and complied with the Declaration of Helsinki.

The following demographic and clinical parameters were obtained from medical records: age, Sex, duration of diabetes history, HbA1c level, Body Mass Index (BMI), abdominal circumference, blood pressure, history of hypertension, serum lipid profile, history of hyperlipidemia, serum uric acid level, serum homocysteine level, history of peripheral atherosclerosis and history of diabetic peripheral neuropathy. Laboratory indexes associated with DN were collected: serum creatinine (Scr) level, urine microalbumin level and urine creatinine level. Estimated glomerular filtration rate (eGFR) was calculated according to previous guidelines (for male: eGFR (ml/min/1.73 m^2) = 186* Scr-1.154*(age)-0.203; for female: eGFR (ml/min/1.73 m^2) = 186*Scr-1.154* (age)- 0.203*0.742). Urine albumin creatine ratio (ACR) was calculated as the urine microalbumin level divided by urine creatinine level. The stage of kidney function was classified as follows: stage I was defined as eGFR at least 90 ml/min/1.73 m^2 as, stage II was defined as eGFR at least 60 ml/min/1.73 m^2 but less than 90 ml/min/1.73 m^2, stage III was defined as eGFR at least 30 ml/min/1.73 m^2 but less than 60 ml/min/1.73 m^2, stage IV was defined as eGFR at least 15 ml/min/1.73 m^2 but less than 30 ml/min/1.73 m^2, while stage V was defined as eGFR less than 15 ml/min/1.73 m^2. The stage of urine microalbumin was classified as follows: stage I was defined as ACR less than 30 mg/g, stage II was defined as ACR at least 30 mg/g but less than 300 mg/g, while stage III was defined as ACR at least 300 mg/g.

All patients had a throughout ophthalmic examination, including best corrected visual acuity (BCVA) using a Snellen chart, intraocular pressure (IOP) measurement, slit lamp examination, color fundus photography, optical coherence tomography (OCT, SS-OCT or SD-OCT, Topcon, Tokyo, Japan) and fluorescein angiography (FFA) if needed. Visual impairment was graded according to BCVA in Snellen chart as: mild, moderate, severe and blind. BCVA ≥ 0.8 was defined as normal vision; 0.6 ≤ BCVA < 0.8 was defined as mild visual impairment; 0.3 ≤ BCVA < 0.6 was defined as moderate visual impairment; 0.1 ≤ BCVA < 0.3 was defined as severe visual impairment; BCVA < 0.1 was defined as blind.

The presence of DR was examined by general ophthalmologists at Department of Ophthalmology and was re-examined by one of our authors YYJ, an experienced retinal specialist, according to the color fundus image, OCT and FFA. The stage of DR was graded according to literature report [14] as: absent DR, mild non-proliferative DR (NPDR), moderate NPDR, severe NPDR, and proliferative diabetic retinopathy (PDR). Absent DR means there is no apparent retinopathy; mild NPDR refers to microaneurysms only. The risk of significant progression over several years is very low in the above groups; moderate NPDR refers to more than just microaneurysms but less than severe NPDR; severe NPDR carries with it the most ominous prognosis for progression to PDR. The lower threshold for entry into this category was the presence of any lesions consistent with the 4:2:1 rule (more than 20 intraretinal hemorrhages in each of 4 quadrants; definite venous beading in 2 quadrants; Prominent intraretinal microvascular abnormalities in 1quadrant and no signs of proliferative retinopathy). PDR includes all eyes with definite neovascularization, vitreous/preretinal hemorrhage, or retinal detachment. Severe NPDR, PDR, and DME with any DR were defined as sight threatening DR. The eye with more advanced DR was selected for grading and analysis in this study.

The presence of HEs was made by visible white-yellow exudates in the posterior area in the color fundus images. Confirmation was made by cross-sectional OCT images as hyperreflective round elements in the outer plexiform layer of the retina. Macular edema was defined as hard exudates in the presence of microaneurysms and blot hemorrhage within 1500 um from the foveal center with a central retinal thickness (CRT) ≥ 250 μm due to intraretinal fluid or subretinal fluid [14]. Macular edema was confirmed based on OCT images.

### Statistics

All statistical analyses were carried out with SPSS software (version 12.0, SPSS, Chicago, IL). Data are expressed as mean ± SD or medians (interquartile range, IQR) for continuous variable and as numbers (percentage) for categorical variables. The t-test was used to compare continuous variables with normal distribution and Mann–Whitney test was used to compare continuous variables which were not normally distributed. The numbers of categorical variables were compared using chi-square test or Fisher’s exact test. Multivariate logistic regression analysis was performed to assess the independent predictive effects of the variables on the risk for progression of DR. *P* values less than 0.05 were considered to indicate statistical significance.

## Results

Records of 145 patients with type 2 DM and history of DN were examined. The duration of DM was 15.14 ± 8.56 years (Table 1). Demographic and clinical characteristics of patients were summarized in Table1. Five patients were excluded for lacking of ophthalmological evaluations. There were 140 eyes of 140 patients included for analyzing with 86 (61.4%) of them having DR. PDR accounted for 23.6% (33 eyes) in the 140 eyes and for 38.4% in DR eyes. Sight threatening DR were accounted for 35.7% (50 eyes) in the 140 eyes and for 58.1% in DR eyes, including SNPDR 8 eyes, PDR 33 eyes and DME with any DR 9 eyes.

**Table 1:** General characteristics of the study population

Variable	
Sex, N (%)	
Male	88/145 (60.7)
Female	57/145 (39.3)
Age, years, mean ± SD	60.35 ± 12.70
Duration of DM, years, mean ± SD	15.14 ± 8.56
BMI, kg/m^2^, years, mean ± SD	26.34 ± 4.00
Abdominal circumference, cm, mean ± SD	95.74 ± 12.58
Systolic pressure, mmHg, mean ± SD	141.92 ± 21.58
Diastolic pressure, mmHg, mean ± SD	79.57 ± 11.31
Stage of kidney function, N (%)	
Stage 1	52/143 (36.4)
Stage 2	43/143 (30.1)
Stage 3	37/143 (25.9)
Stage 4	5/143 (3.5)
Stage 5	6/143 (4.2)
Stage of urine microalbumin, N (%)	
Stage 1	12/124 (9.7)
Stage 2	52/124 (41.9)
Stage 3	60/124 (48.4)
DR severity scale, N (%)	
None	54/140 (38.6)
Mild NPDR	11/140 (7.9)
Moderate NPDR	34/140 (24.3)
SNPDR	8/140 (5.7)
PDR	33/140 (23.6)
Visual impairment, N (%)	
Normal	20/133 (15.0)
Mild	49/133 (36.8)
Moderate	41/133 (30.8)
Sever	18/133 (13.5)
Blind	5/133 (3.8)

There were missing values in kidney function (2 missing), urine microalbumin (16 missing) and DR severity (5 missing) and visual impairment (12 missing). There were 35 eyes with cataract and 12 eyes with intraocular lends implantation

*DM* Diabetes mellitus, *BMI* Body mass index, *DR* Diabetic retinopathy, *PDR* Proliferative diabetic retinopathy, *NPDR* Non-proliferative diabetic retinopathy, *SNPDR* Sever non-proliferative diabetic retinopathy

Analysis of laboratory profiles found no significant difference between DR group and non-DR group in the levels of triglycerides, total and high-density lipoprotein cholesterol (HDL-C), serum uric acid, serum homocysteine (HCY), serum creatinine. However, DR group had significantly higher levels of low-density lipoprotein cholesterol (LDL-C) (*p* = 0.004), HbA1c (*P* = 0.037), ACR(*p* < 0.001) and lower level of eGFR(*P* = 0.013). (Table 2).

**Table 2:** Demographic and clinical characteristics in DR and Non-DR groups

Variable	DR *N* = 86	Non-DR *N* = 54	*P* value
Sex, N (%)			0.038^*^
Male	47/86 (54.7)	39/54 (72.2)	
Female	39/86 (45.3)	15/54 (27.8)	
Age, years, mean ± SD	58.78 ± 12.15	62.65 ± 13.14	0.078
BMI, kg/m^2^, mean ± SD	26.16 ± 3.78	26.74 ± 4.36	0.412
Abdominal circumference, cm, mean ± SD	94.653 ± 12.25	98.29 ± 12.59	0.169
Systolic pressure, mmHg, mean ± SD	144.40 ± 28.36	137.39 ± 26.75	0.149
Diastolic pressure, mmHg, mean ± SD	80.71 ± 11.43	77.27 ± 11.23	0.126
Stage of hypertension, N (%)			0.624
Normal blood pressure	15/85 (17.6)	12/54 (22.2)	
Stage 1	21/85 (24.7)	17/54 (31.5)	
Stage 2	15/85 (17.6)	7/54 (13.0)	
Stage 3	34/85 (40.0)	18/54 (33.3)	
Serum lipid profiles			
Total cholesterol, mmol/L, mean ± SD	4.60 ± 1.60	4.13 ± 1.29	0.063
Triglycerides, mmol/L, mean ± SD	2.51 ± 2.88	2.76 ± 4.39	0.681
HDL cholesterol, mmol/L, mean ± SD	1.08 ± 0.51	1.02 ± 0.36	0.523
LDL cholesterol, mmol/L, mean ± SD	2.84 ± 1.02	2.40 ± 0.72	0.004^*^
History of hyperlipidemia, N (%)			0.044^*^
No	31/85 (36.5)	11/54 (20.4)	
Yes	54/85 (63.5)	43/54 (79.6)	
History of hyperuricemia, N (%)			0.569
No	68/85 (80.0)	41/54 (75.9)	
Yes	17/85 (20.0)	13/54 (24.1)	
History of hyperhomocystinemia, N (%)			0.843
No	75/85 (88.2)	47/54 (87.0)	
Yes	10/85 (11.8)	7/54 (13.0)	
Serum uric acid, μmol/L, mean ± SD	361.15 ± 115.61	367.81 ± 90.01	0.725
Serum HCY, mmol/L, mean ± SD	15.89 ± 7.38	15.16 ± 6.50	0.581
Duration of DM, years, mean ± SD	14.80 ± 7.73	15.21 ± 9.58	0.781
HbA1c, %, mean ± SD	9.07 ± 2.27	8.30 ± 1.62	0.037^*^
ACR, mg/g, median (interquartile range)	630.82 (186.02, 1806.13)	64.13 (31.56, 327.96)	< 0.001^*^
Stage of urine microalbumin, N (%)			< 0.001^*^
Stage 1	1/70 (1.4)	11/50 (22.0)	
Stage 2	26/70 (37.1)	25/50 (50.0)	
Stage 3	43/70 (61.4)	14/50 (28.0)	
Serum creatinine, μmol/L, median (interquartile range)	91.30 (66.38, 125.35)	78.25 (62.93, 106.70)	0.091
eGFR, ml/min/1.73m^2^, median (interquartile range)	74.47 (44.48, 97.84)	89.60 (58.76, 102.45)	0.013^*^
Stage of kidney function, N (%)			/
Stage 1	26/84 (31.0)	25/54 (46.3)	
Stage 2	27/84 (32.1)	14/54 (26.0)	
Stage 3	20/84 (23.8)	15/54 (27.8)	
Stage 4	5/84 (6.0)	0/54 (0.0)	
Stage 5	6/84 (7.1)	0/54 (0.0)	
History of peripheral atherosclerosis, N (%)			0.185
No	46/86 (53.5)	23/54 (42.6)	
Yes	40/86 (46.5)	31/54 (57.4)	
History of diabetic peripheral neuropathy, N (%)			0.682
No	37/83 (44.6)	26/54 (48.1)	
Yes	46/83 (55.4)	28/54 (51.9)	

There were missing values in stage of hypertension (1 missing), history of hyperlipidemia (1 missing), history of hyperuricemia (1 missing), history of hyperhomocystinemia (1 missing), stage of urine microalbumin (20 missing), stage of kidney function (2 missing) and history of diabetic peripheral neuropathy (3 missing). Data are displayed as means ± SD, N (%), or median (interquartile range) and compared by Student t-test, Mann-Whiteney U test or chi-square test where appropriate. * indicates *p* value < 0.05

*DR* Diabetic retinopathy, *HDL* High-density lipoprotein cholesterol, *LDL* Low-density lipoprotein cholesterol, *HCY* serum homocysteine, *ACR* Urine albumin creatine ratio

In a logistic regression analysis controlled for sex, LDL-C, HbA1c and eGFR, DR was significantly associated with ACR stage (*p* = 0.011). Subjects with ACR stage3 had higher incidence of DR compared with subjects with ACR stage1 (OR = 24.15. 95%CI: 2.06–282.95) (Table 3).

**Table 3:** Risk factor of DR in DM patients with DN

Variable	OR	95% CI	*P* value
Sex			
Male	Reference		
Female	3.94	1.38–11.30	0.011
ACR			
Stage1	Reference		
Stage2	6.16	0.61–61.80	0.122
Stage3	24.15	2.06–282.95	0.011

Variables with p values less than 0.05 are included in the Logistic analysis

*DR* Diabetic retinopathy, *DM* Diabetes mellitus, *DN* Diabetic nephropathy, *ACR* Urine albumin creatine ratio

Seven of the patients lacked clear OCT images. So, there were 138 eyes of 138 patients who had clear OCT images analyzed for HEs and DME. 32 eyes (23.2%) had HEs in the area of posterior pole and 13 eyes (9.4%) had DME. There was significant difference in the LDL-C level, total cholesterol level and ACR between HEs group and non-HEs group (*p* = 0.008, *p* = 0.014, *p* = 0.001 respectively). Visual acuity was worse in HEs group than in non-HEs group, however, no significant difference was found between the groups(*p* = 0.058) (Table 4). Similarly, there was significant difference in the LDL-C level, and ACR between DME group and non-DME group (*p* = 0.020, *p* = 0.009 respectively). Visual acuity was worse in DME group with significant difference compared with non-DME group (*p* = 0.003) (Additional file 2).

**Table 4:** Demographic and clinical characteristics in HEs and Non-HEs groups

Variable	HEs *N* = 32	Non-HEs *N* = 106	*P* value
Sex, N (%)			0.018^*^
Male	14/32 (43.7)	71/106 (67.0)	
Female	18/32 (56.3)	35/106 (33.0)	
Age, years, mean ± SD	59.91 ± 11.11	60.45 ± 13.14	0.831
Visual acuity, LogMAR, mean ± SD	0.91 ± 0.75	0.63 ± 0.69	0.058
IOP, mmHg, mean ± SD	14.29 ± 3.41	15.04 ± 3.72	0.338
BMI, kg/m^2^, mean ± SD	25.81 ± 3.55	26.60 ± 4.10	0.330
Abdominal circumference, cm, mean ± SD	93.26 ± 10.48	96.82 ± 12.91	0.273
Systolic pressure, mmHg, mean ± SD	147.44 ± 27.31	140.26 ± 28.06	0.204
Diastolic pressure, mmHg, mean ± SD	77.31 ± 10.08	80.29 ± 11.73	0.171
Stage of hypertension, N (%)			0.598
Normal blood pressure	6/32 (18.8)	21/105 (20.0)	
Stage 1	6/32 (18.8)	30/105 (28.6)	
Stage 2	7/32 (21.9)	15/105 (14.3)	
Stage 3	13/32 (40.6)	39/105 (37.1)	
Serum lipid profiles			
Total cholesterol, mmol/L, mean ± SD	5.01 ± 1.52	4.25 ± 1.47	0.014^*^
Triglycerides, mmol/L, mean ± SD	3.34 ± 4.20	2.37 ± 3.32	0.181
HDL cholesterol, mmol/L, mean ± SD	1.04 ± 0.24	1.06 ± 0.50	0.848
LDL cholesterol, mmol/L, mean ± SD	3.06 ± 0.85	2.55 ± 0.94	0.008^*^
History of hyperlipidemia, N (%)			0.007^*^
No	16/32 (50.0)	26/105 (24.8)	
Yes	16/32 (50.0)	79/105 (75.2)	
History of hyperuricemia, N (%)			0.623
No	26/32 (81.3)	81/105 (77.1)	
Yes	6/32 (18.8)	24/105 (22.9)	
History of hyperhomocystinemia, N (%)			0.359
No	30/32 (93.8)	90/105 (85.7)	
Yes	2/32 (6.3)	15/105 (14.3)	
Serum uric acid, μmol/L, mean ± SD	385.45 ± 113.09	358.75 ± 103.30	0.220
Serum HCY, mmol/L, mean ± SD	14.55 ± 4.38	16.00 ± 7.71	0.338
Duration of DM, years, mean ± SD	13.89 ± 7.02	15.19 ± 8.92	0.450
HbA1c, %, mean ± SD	9.07 ± 2.14	8.86 ± 2.03	0.322
ACR, mg/g, median (interquartile range)	688.28 (222.59, 3455.69)	194.83 (47.12, 758.14)	0.001^*^
Stage of urine microalbumin, N (%)			0.056
Stage 1&2	9/25 (36.0)	54/94 (57.4)	
Stage 3	16/25 (64.0)	40/94 (42.6)	
Serum creatinine, μmol/L, median (interquartile range)	92.70 (68.50, 104.40)	81.40 (63.45, 113.65)	0.490
eGFR, ml/min/1.73m^2^, mean ± SD	66.55 ± 29.94	77.70 ± 29.03	0.064
Stage of kidney function, N (%)			0.157
Stage 1	8/31 (25.8)	42/105 (40.0)	
Stage 2	11/31 (35.5)	30/105 (28.6)	
Stage 3	7/31 (22.6)	28/105 (26.7)	
Stage 4	2/31 (6.5)	3/105 (2.9)	
Stage 5	3/31 (9.7)	2/105 (1.9)	
History of peripheral atherosclerosis, N (%)			0.962
No	16/32 (50.0)	52/105 (49.5)	
Yes	16/32 (50.0)	53/105 (50.5)	
History of diabetic peripheral neuropathy, N (%)			0.683
No	15/31 (48.4)	46/104 (44.2)	
Yes	16/31 (51.6)	58/104 (55.8)	

There were missing values in stage of hypertension (1 missing), history of hyperlipidemia (1 missing), history of hyperuricemia (1missing), history of hyperhomocystinemia (1 missing), stage of urine microalbumin (19 missing), stage of kidney function (2 missing), history of peripheral atherosclerosis (1 missing) and history of diabetic peripheral neuropathy (3 missing). Data are displayed as means ± SD, N (%), or median (interquartile range) and compared by Student t-test, Mann-Whiteney U test or chi-square test where appropriate. * indicates *p* value < 0.05

*IOP* Intraocular pressure, *DR* Diabetic retinopathy, *HDL* High-density lipoprotein cholesterol, *LDL* Low-density lipoprotein cholesterol, *HCY* Serum homocysteine, *ACR* Urine albumin creatine ratio

## Discussion

DR is a kind of serious microvascular complication of type 2 DM, causing visual impairment in adults. In order to investigate the factors associated with DR, we collected demographic and clinical information of diabetic patients with history of DN and analyzed the differences of these factors between DR group and non-DR group. we found that DR group had significantly higher levels of LDL-C, HbA1c, ACR, and lower levels of eGFR compared with non-DR group. Further logistic regression analysis indicated that higher stage of ACR was significantly associated with DR.

The retina and the kidney complications of DM both result from damage to small vessels in these organs. The prevalence of DR among type 2 DM was among 20%-30% in previous population-based studies. In Korea, the prevalence of DR and PDR among type 2 DM patients were 20.0% and 3.8% [15]. In a nationally representative sample of US adults with diabetes more than 40 years old, the prevalence of DR and PDR were 28.5% and 1.5% [16]. The prevalence of DR was 22.1% in diabetic patients aged 50–59 years in China [3].

Our study revealed that the DN patients exhibited relatively high prevalence of both DR and PDR. This was consistent with previous studies that DN patients were more likely to have DR [6, 15, 17–19]. In our hospital-based study, we found that the prevalence of DR and PDR among type 2 DM patients with history of DN were 61.4% and 23.6% respectively, which were much higher than those in type 2 DM patients in previous hospital-based studies. In a retrospective study in Lanzhou, DR was present in 48.8% in type 2 DM patients with confirmed diabetic nephropathy [18]. In a prospective cohort study in Taiwan, the prevalence of DR and PDR among type 2 DM patients with a mean urinary ACR of 92.2 ± 64.0 mg/g (range: 30–299 mg/g) were 43.4% and 2.4% [20]. In our study, 48.4% of patients had urinary ACR at least 300 mg/g and 41.9% had urinary ACR between 30 mg/g and 299 mg/g. The reason why our study found much higher prevalence of DR and PDR may be due to that we enrolled more patients with severe renal impairment. Based on the above results, we believe that besides routine ophthalmic examination in patients with type 2 DM, patients with DN needs ophthalmic examination more timely and more frequently.

Microalbuminuria is identified as an early sign of DN. Logistic regression analysis in our study showed that subjects with ACR stage3 had higher incidence of DR compared with subjects with ACR stage1(OR = 24.15, 95%CI: 2.06–282.95) (Table2&3). Chen et al. found similar results that high urinary albumin levels were associated with more serious DR [21]. A five-year hospital-based cohort study of 462 patients in America reported that microalbuminuria was independently associated with the incidence and progression of DR [22]. A prospective cross-sectional study of 306 patients over a period of 2 years also revealed that microalbuminuria was strong predictor for DR [23]. Lee et al. also found PDR was associated with microalbuminuria [15].The reason why DR is associated with microalbuminuria has not been clearly demonstrated. It has been reported that more serious endothelial cell damage appeared in hypertensive subjects with microalbuminuria [24]. Moreover, intercellular adhesion molecule (ICAM) and E-selectin were 10.2% and 15.5% higher respectively in adolescent participants with microalbuminuria and type 2 DM [25]. Besides, in an animal model of diabetic kidney disease (DKD), marked expression of vascular endothelial growth factor (VEGF) was observed in reaction to glomerular injury [26]. Elevated serum VEGF levels have also been noted in patients with chronic renal failure [27]. Thus, DKD may facilitate the development and progression of DR due to overproducing VEGF, which may then reach the eyes via systemic circulation. These all give evidence that endothelial dysfunction together with elevation circulating adhesion molecules and VEGF may be the possible mechanism for DR formation or progression in DN patients.

Macular HEs are often seen in patients with diabetic macular edema (DME) and they are known to be an independent risk factor for vision loss [11, 28]. Even eventual disappearance of HEs is not necessarily followed by improvement in visual acuity. We found significant difference in ACR between HEs group and non-HEs group (Table 4), which implies possible association between renal function and HEs. Toshihiko [13] reported disappearance of HEs after introduction of hemolysis in chronic renal failure patients caused by DN. The reason why renal function level is associated with HEs has not been clearly demonstrated. One of the possible mechanisms may be that renal function impairment could result in damage of renal vascular cells as well as retinal pigment epithelial cells. These could cause blood-retinal barrier damage and lead to formation of HEs. We also found difference in the LDL-C level, total cholesterol level between HEs group and non-HEs group. Previous studies have also found similar results that there were associations between higher total or/and LDL-C levels and HEs [11,29, 30], but the results vary in glycerin triglyceride (TG). Sachdev et al. [31] and Sasaki et al. [32] found association with TG and HEs, while in our study, we found no statistical difference in TG between HEs group and non-HEs group. The variation between the results in our study and previous studies could be due to differences in HEs assessment methods and age, sex, diabetes duration, diabetes type, ethnicity etc.

Our study has several limitations. Firstly, the present study was a retrospective cross-sectional study, thus causal relationships cannot be established. Secondly, the study was conducted in hospitalized patients in one center, thus our results may not be representative to other populations. Further prospective study in multi-center is needed in the future to prove the association between urine microalbumin and DR.

## Conclusions

In conclusion, a relatively higher prevalence of DR was found in type 2 DM patients with DN. DM was significantly associated with ACR stage, thus ACR stage could be recognized as a risk factor for DR in DN patients. Besides routine ophthalmic examination in patients with type 2 DM, patients with DN needs ophthalmic examination more timely and more frequently. These data may provide useful information for the prevention and management of DM complications.

## Supplementary Information

**Additional file 1.**

**Additional file 2.** 

## Data Availability

Data is available in the supplementary materials.

## Abbreviations

ACR: Urine albumin creatine ratio
BMI: Body Mass Index
BCVA: Best corrected visual acuity
CHOL: Total cholesterol
CRT: Central retinal thickness
DN: Diabetic nephropathy
DM: Diabetic mellitus
DR: Diabetic retinopathy
DKD: Diabetic kidney disease
DME: Diabetic macular edema
eGFR: Estimated glomerular filtration rate
FFA: Fluorescence angiography
HEs: Hard exudates
HDL-C: High-density lipoprotein cholesterol
HCY: Serum homocysteine
IQR: Interquartile range
ICAM: Intercellular adhesion molecule
IOP: Intraocular pressure
LDL-C: Low-density lipoprotein cholesterol
NPDR: Non-proliferative DR
OCT: Optical coherence tomography
PDR: Proliferative diabetic retinopathy
Scr: Serum creatinine
TG: Glycerin triglyceride
VEGF: Vascular endothelial growth factor
