# Supplementary material for: Retinal microvascular changes in diabetic patients with diabetic nephropathy
Source: BMC Endocr Disord. 2023 May 5;23:101. doi: 10.1186/s12902-022-01250-w (PMC10161482; doi:10.1186/s12902-022-01250-w)
Supplement: Supplementary file 1 — Additional file 1. [file 12902_2022_1250_MOESM1_ESM.pdf]

	No	ID	Sex	Age	HBPstage	HBP	HBPyears
1	1	703576	2	66	2	1	22.0
2	2	935812	1	61	3	1	10.0
3	3	11137325	1	73	3	1	27.0
4	4	1161428	1	55	0	0	.0
5	5	1183488	1	53	2	1	10.0
6	6	1189287	2	61	3	1	10.0
7	7	1392578	1	65	0	0	.0
8	8	1407883	1	74	2	1	6.0
9	9	2205884	2	64	3	1	10.0
10	10	2264369	1	45	0	0	.0
11	11	2264627	2	51	2	1	5.0
12	12	2265205	1	54	1	1	6.0
13	13	2267377	1	54	0	0	.0
14	14	2267629	1	58	1	1	3.0
15	15	2267756	1	40	2	1	14.0
16	16	2269945	2	24	1	1	1.0
17	17	1271278	1	45	0	0	.0
18	18	1286587	2	50	0	0	.0
19	19	1339842	2	21	0	0	.0
20	20	1430377	1	59	1	1	15.0
21	21	1463410	2	56	0	0	.0
22	22	1491140	2	66	3	1	5.0
23	23	184601	2	76	1	1	18.0
24	24	1187651	1	82	1	1	20.0
25	25	1374614	2	79	3	1	6.0
26	26	1388851	2	64	0	0	.0
27	27	1457789	2	46	0	0	.0
28	28	1469520	2	60	3	1	20.0
29	29	1747086	2	85	3	1	15.0
30	30	2231976	1	70	1	1	.
31	31	2270046	1	56	3	1	1.0
32	32	2271261	1	67	1	1	50.0
33	33	2271829	1	54	3	1	15.0
34	34	982864	1	68	2	1	10.0
35	35	1379985	2	62	3	1	10.0
36	36	1408233	2	33	2	1	10.0
37	37	1479683	1	35	1	1	2.0
38	38	2236182	1	69	1	1	1.0
39	39	2274609	1	70	3	1	16.0

	CHD	PA	PN	UA	HCY	DNstage	DNstageadjust
1	0	1	0	0	0	1	1
2	1	0	1	0	0	5	3
3	0	0	1	0	0	1	1
4	0	0	0	0	1	3	4
5	1	0	1	0	0	4	4
6	0	0	1	0	0	4	3
7	0	1	0	0	0	3	4
8	0	0	0	0	0	3	4
9	0	0	1	0	0	4	4
10	0	1	1	0	0	1	1
11	0	0	1	0	0	.	3
12	0	0	0	0	0	3	3
13	0	1	0	1	0	3	3
14	0	1	1	1	0	1	3
15	0	1	0	0	0	2	3
16	0	0	1	0	0	2	3
17	1	0	1	0	1	3	4
18	0	1	1	0	0	3	3
19	0	0	0	1	0	3	3
20	0	1	1	0	0	3	3
21	0	0	1	0	0	4	2
22	0	1	0	1	1	3	4
23	1	0	1	1	0	.	3
24	1	1	0	0	0	4	4
25	0	0	0	0	0	3	4
26	0	1	0	0	0	3	3
27	0	1	1	0	0	4	4
28	0	1	0	0	0	3	3
29	0	0	0	1	0	4	4
30	1	0	0	0	0	3	3
31	1	1	1	0	0	4	4
32	1	0	1	1	0	4	4
33	0	0	1	1	0	3	2
34	1	1	1	0	0	1	3
35	1	1	1	0	0	4	4
36	0	1	1	1	0	3	3
37	0	1	0	0	0	1	3
38	0	0	1	0	1	3	4
39	0	1	1	0	0	2	3

	DNstage2	DNstage3	KDeGFR	UPstage	UPstage2	HL
1	1	1	1	1	1	0
2	1	2	3	2	1	1
3	1	1	1	1	1	0
4	2	3	1	3	2	1
5	2	3	1	3	2	1
6	1	2	3	.	.	1
7	2	3	3	3	2	0
8	2	3	2	3	2	1
9	2	3	2	3	2	0
10	1	1	1	1	1	0
11	1	2	2	2	1	0
12	1	2	1	2	1	1
13	1	2	2	2	1	1
14	1	2	1	2	1	1
15	1	2	2	2	1	1
16	1	2	2	2	1	0
17	2	3	3	3	2	1
18	1	2	1	2	1	1
19	1	2	1	2	1	0
20	1	2	2	2	1	1
21	1	1	2	.	.	0
22	2	3	2	3	2	1
23	1	2	3	1	1	1
24	2	3	3	3	2	1
25	2	3	3	3	2	0
26	1	2	1	2	1	1
27	2	3	1	3	2	0
28	1	2	3	2	1	1
29	2	3	4	3	2	1
30	1	2	2	2	1	0
31	2	3	1	3	2	0
32	2	3	2	3	2	1
33	1	1	2	.	.	1
34	1	2	1	2	1	1
35	2	3	3	3	2	1
36	1	2	1	2	1	1
37	1	2	1	2	1	1
38	2	3	3	3	2	0
39	1	2	2	2	1	1

	HLyears	HLmedicine	DMtype	DMyears
1	.00	0	2	22.0
2	6.00	0	2	8.0
3	.00	0	2	15.0
4	.00	0	2	4.0
5	10.00	1	2	7.0
6	.	0	2	29.0
7	.00	0	2	18.0
8	6.00	1	2	19.0
9	.00	0	2	22.0
10	.00	0	2	13.0
11	.00	0	2	11.0
12	2.00	0	2	4.0
13	.00	0	2	10.0
14	5.00	0	2	15.0
15	10.00	0	2	14.0
16	.00	0	2	10.0
17	10.00	0	2	20.0
18	.00	0	2	13.0
19	.00	0	1	13.0
20	.00	0	2	17.0
21	.00	0	2	14.0
22	2.00	0	2	6.0
23	.00	0	2	20.0
24	.00	0	2	12.0
25	.00	0	2	20.0
26	7.00	1	2	17.0
27	.00	0	2	6.0
28	10.00	1	2	8.0
29	4.00	0	2	10.0
30	.00	0	2	12.0
31	.00	0	2	8.0
32	.00	0	2	12.0
33	.00	0	2	15.0
34	32.00	1	2	32.0
35	6.00	1	2	15.0
36	.10	0	2	8.0
37	.00	0	2	14.0
38	.00	0	2	20.0
39	4.00	1	2	28.0

	HbA1c	Height	WeightKg	BMI
1	7.30	170	70.0000	24.2200
2	7.30	170	80.0000	.
3	7.20	175	90.0000	29.3900
4	9.40	175	71.7000	23.4000
5	10.80	173	83.3500	27.8400
6	8.40	165	84.4000	31.0000
7	6.90	182	79.8000	24.0000
8	10.00	180	72.7000	22.4000
9	9.80	164	80.0000	29.7000
10	7.70	180	91.0000	28.0900
11	.	158	58.0000	23.2000
12	7.90	171	96.0000	33.2200
13	10.60	180	90.0000	27.7800
14	8.10	170	67.9000	23.5000
15	7.60	173	99.6000	33.2800
16	6.90	170	68.5500	23.7000
17	9.40	170	75.0000	25.9500
18	11.30	155	61.0000	25.3900
19	10.10	163	59.0000	21.0000
20	12.00	172	96.3000	32.5100
21	7.10	170	81.0000	27.6800
22	.	171	88.2000	30.1600
23	.	172	77.3000	26.1000
24	10.00	169	80.0000	28.0000
25	10.80	162	72.0000	27.4300
26	6.60	149	50.0000	22.5000
27	9.90	163	85.0000	32.0000
28	11.70	160	108.0000	42.1800
29	7.40	158	67.0000	26.8400
30	7.40	166	60.0000	21.7800
31	11.80	170	69.0000	23.8800
32	9.50	176	67.0000	21.0000
33	.	170	91.0000	30.8000
34	12.50	171	80.0000	27.4000
35	10.40	154	61.0000	25.7000
36	8.00	177	98.0000	31.2800
37	.	180	96.0000	29.6000
38	7.80	170	73.0000	25.3000
39	7.60	160	55.8000	21.7900

	AC	SBP	DBP	SCRumolL
1	.	117	71	75.10
2	.	134	90	115.30
3	.	140	73	55.30
4	88.0	134	92	70.50
5	91.0	141	90	63.80
6	.	145	58	109.40
7	103.0	135	75	113.10
8	102.0	153	86	69.20
9	.	160	79	84.00
10	.	120	70	54.60
11	84.3	120	80	75.10
12	.	120	80	53.00
13	.	141	90	84.80
14	93.5	141	88	70.30
15	115.0	132	91	100.70
16	91.0	94	67	86.10
17	95.0	123	78	124.90
18	.	120	80	49.60
19	70.0	20	80	52.80
20	109.0	147	87	100.10
21	.	155	82	93.10
22	105.0	168	93	87.90
23	98.0	157	68	142.50
24	.	122	76	157.40
25	95.0	225	81	102.50
26	84.0	156	76	61.80
27	.	156	85	37.70
28	128.0	196	79	105.80
29	.	185	100	233.90
30	.	170	80	77.10
31	103.0	160	95	47.10
32	.	175	89	80.70
33	135.0	135	75	88.30
34	102.3	133	78	39.20
35	95.0	149	71	95.40
36	.	175	103	66.60
37	92.0	135	86	70.00
38	96.0	125	64	140.80
39	82.5	145	64	94.70

renamed_63cd7 12.10 submit.sav

	eGFRmlmin1.73m2	CKDstage	DKDstage	UPstageadj	UAadj
1	90.94	1	1	1	415.00
2	58.90	3	3	2	434.00
3	98.18	1	1	1	286.00
4	100.48	1	4	3	358.00
5	106.55	1	4	3	308.00
6	47.81	3	3	.	462.00
7	58.61	3	4	3	445.00
8	88.91	2	4	3	240.00
9	63.09	2	4	3	342.00
10	112.13	1	1	1	383.00
11	83.71	2	3	2	255.00
12	115.80	1	3	2	385.00
13	89.69	2	3	2	430.00
14	98.85	1	3	2	475.00
15	80.40	2	3	2	358.00
16	81.68	2	3	2	254.00
17	59.83	3	4	3	332.00
18	108.77	1	3	2	227.00
19	134.27	1	3	2	446.00
20	76.46	2	3	2	418.00
21	73.35	2	2	.	333.00
22	78.94	2	4	3	432.00
23	41.03	3	3	1	523.00
24	35.28	3	4	3	394.00
25	55.81	3	4	3	410.00
26	91.70	1	3	2	341.00
27	122.44	1	4	3	254.00
28	46.09	3	3	2	315.00
29	17.17	4	4	3	485.00
30	84.47	2	3	2	437.00
31	118.18	1	4	3	399.00
32	86.92	2	4	3	546.00
33	85.41	2	2	.	.
34	113.15	1	3	2	283.00
35	55.25	3	4	3	265.00
36	115.50	1	3	2	609.00
37	113.96	1	3	2	426.00
38	43.73	3	4	3	4.90
39	70.14	2	3	2	416.00

	ACRmgmmol	ACRmgg	UP24hg	CHOLmmolL	TGmmolL
1	1.16	10.25	.19	2.10	1.09
2	3.97	35.09	.	4.27	2.60
3	.30	2.65	.	2.81	1.33
4	47.51	419.99	.	5.23	6.26
5	71.32	630.47	1.93	3.08	3.33
6	.	.	.43	4.04	1.46
7	84.82	749.81	.55	3.98	.91
8	83.89	741.59	1.23	4.07	1.67
9	172.72	1526.84	.	6.72	4.00
10	.55	4.86	.	3.76	2.95
11	31.71	280.32	.	5.32	1.22
12	11.18	98.83	.06	3.54	3.90
13	3.82	33.77	.46	4.56	2.48
14	11.33	100.16	.	2.84	2.44
15	4.38	38.72	.19	3.24	1.06
16	19.08	168.67	.	3.65	.90
17	89.16	788.17	.	2.77	2.53
18	25.71	227.28	.27	4.25	1.53
19	27.28	241.16	.	3.84	.71
20	12.67	112.00	.	4.21	1.57
21	.	.	5.86	4.75	1.38
22	62.03	548.35	.	10.46	32.78
23	.62	5.48	.05	4.21	1.59
24	169.52	1498.56	2.77	3.49	1.20
25	70.36	621.98	2.39	4.14	1.30
26	6.40	56.58	.	3.53	1.27
27	84.71	748.84	1.64	6.95	2.75
28	20.32	179.63	.	4.56	1.52
29	250.41	2213.62	2.22	6.61	2.38
30	20.33	179.72	.18	4.30	1.10
31	88.59	783.14	.67	4.81	4.87
32	202.63	1791.25	1.61	6.85	2.08
33	.	.	.	.	.
34	11.21	99.10	.	3.00	1.77
35	55.26	488.50	.	2.39	1.08
36	5.63	49.77	.14	6.67	3.83
37	6.77	59.85	.	5.08	5.19
38	556.04	4915.39	2.10	2.61	.72
39	26.14	231.08	.	3.74	.84

	HDLCmmolL	LDLCmmolL	HCYumolL	GLUmmolL
1	.78	1.18	.	.
2	.84	2.77	18.27	.
3	.82	1.62	19.45	.
4	.89	2.64	.	10.72
5	.77	1.90	.	9.86
6	.92	2.56	15.34	5.28
7	.97	2.57	.	9.33
8	.91	2.64	18.61	11.79
9	1.18	4.26	.	.
10	.80	2.43	.	.
11	1.23	3.40	11.94	8.56
12	.87	2.34	23.25	8.96
13	.85	3.01	13.52	9.72
14	.71	1.72	10.94	11.06
15	.92	2.06	.	4.71
16	1.07	2.03	13.96	4.31
17	.77	1.55	15.95	5.03
18	1.63	2.64	12.84	8.60
19	1.22	2.25	10.21	10.32
20	.99	2.42	13.47	15.35
21	.98	3.11	12.65	.
22	2.38	.97	13.37	8.99
23	.85	2.65	17.98	6.08
24	.97	2.00	7.45	.
25	1.18	2.73	13.82	9.17
26	1.48	1.92	7.20	7.10
27	1.08	4.63	8.01	13.02
28	1.10	2.76	45.46	8.46
29	1.18	4.29	.	9.71
30	.87	2.96	13.95	5.60
31	1.18	2.80	14.77	12.73
32	1.04	4.32	16.55	.
33	.	.	.	8.37
34	.85	1.75	8.50	10.11
35	.80	1.18	15.21	10.81
36	1.13	4.27	34.41	8.88
37	1.07	2.85	10.87	11.02
38	.91	1.40	15.64	8.57
39	.84	2.35	14.38	5.14

	INSulUml	CPngml	@0.5hGLU	@0.5hINS
1	.	.	.	.
2	3.25	.	.	.
3	.	.	.	.
4	8.53	2.41	13.49	12.21
5	9.65	2.07	17.70	12.66
6	7.26	1.37	6.65	8.58
7	38.32	3.32	14.51	59.52
8	11.28	1.72	13.22	11.02
9	.	.	.	.
10	4.37	1.60	9.75	8.29
11	17.76	1.65	9.85	14.09
12	28.52	4.67	.	.
13	10.51	2.72	.	.
14	6.27	1.51	14.07	10.13
15	11.18	2.84	9.28	28.23
16	4.80	.93	5.34	7.17
17	15.43	1.55	5.38	14.23
18	.	.	.	.
19	11.97	.06	17.10	51.97
20	16.58	188.00	15.08	13.70
21	.	.	.	.
22	56.29	4.37	11.95	64.56
23	15.94	4.57	10.13	45.93
24	.	.	.	.
25	.97	.84	13.26	5.61
26	8.29	2.39	10.88	35.24
27	13.29	2.45	.	.
28	75.06	3.49	12.44	78.76
29	.	7.56	.	.
30	15.42	3.46	.	.
31	8.54	1.96	15.97	10.17
32	.	.	.	.
33	8.95	2.79	15.08	23.04
34	1.28	.52	13.24	1.78
35	244.70	4.43	14.48	298.00
36	18.82	2.39	.	.
37	4.51	1.13	13.55	9.49
38	8.35	2.07	9.46	11.31
39	3.32	.95	9.91	9.07

	@0.5hCP	@1hGLU	@1hINS	@1hCP
1	.	.	.	.
2	.	15.51	42.43	5.35
3	.	.	.	.
4	2.63	16.79	15.86	3.20
5	2.17	17.90	15.11	2.64
6	1.33	7.22	10.20	1.51
7	4.17	20.69	112.60	6.58
8	1.82	17.98	15.25	2.02
9	.	.	.	.
10	2.07	13.21	20.42	3.39
11	1.83	14.63	16.22	2.08
12	.	13.45	56.61	6.90
13	.	12.98	13.80	2.91
14	1.73	16.81	11.84	2.10
15	3.87	13.94	50.23	5.68
16	1.52	5.40	7.31	1.58
17	1.00	7.96	16.62	1.45
18	.	.	.	.
19	.07	20.20	64.38	.08
20	1.78	22.32	17.32	2.05
21	.	.	.	.
22	4.56	13.93	68.15	4.86
23	5.38	14.77	103.20	9.08
24	.	.	.	.
25	1.02	18.21	8.08	1.23
26	4.18	14.49	52.07	5.76
27	.	14.27	17.69	2.84
28	3.60	15.76	92.96	3.49
29	.	.	.	.
30	.	12.37	35.21	4.19
31	2.14	20.89	11.27	2.33
32	.	.	.	.
33	4.65	21.24	5.85	7.76
34	.67	17.98	2.80	.83
35	4.98	19.34	368.80	5.83
36	.	13.26	19.11	2.72
37	1.76	17.46	9.64	1.92
38	2.27	13.26	27.96	9.79
39	1.57	14.43	10.99	2.05

	@2hGLU	@2hINS	@2hCP	DRdosmesticR
1	.	.	.	0
2	16.60	49.46	7.41	0
3	.	.	.	0
4	18.09	18.00	4.19	0
5	17.30	20.75	3.75	1
6	21.70	25.27	3.08	0
7	20.45	118.30	8.80	0
8	20.08	20.10	3.03	1
9	.	25.43	1.52	4
10	12.20	23.59	5.08	0
11	18.36	25.45	4.26	3
12	15.95	59.01	8.78	0
13	14.50	19.45	3.92	0
14	24.01	24.86	3.43	3
15	14.46	67.83	8.45	3
16	6.42	9.78	1.82	5
17	11.28	26.78	9.05	4
18	.	.	.	0
19	23.30	52.06	.08	3
20	25.39	21.74	2.58	3
21	.	.	.	1
22	17.66	87.15	5.97	0
23	1861.00	172.00	16.61	0
24	.	.	.	.
25	23.72	14.77	2.15	2
26	1.09	91.12	9.83	0
27	17.52	16.82	2.85	1
28	21.92	11.51	4.10	0
29	16.69	.	7.47	4
30	10.75	51.27	6.31	0
31	21.58	12.59	2.78	0
32	.	.	.	4
33	19.00	25.22	7.41	0
34	21.91	4.10	1.30	0
35	24.22	557.50	8.05	2
36	14.13	22.13	3.51	0
37	19.62	8.26	2.04	0
38	15.25	27.95	5.32	6
39	22.54	22.98	4.24	2

	DRinternatioinalR	DRdandiR	DRdosmesticL	DRinternatioinalL
1	0	0	0	0
2	0	0	0	0
3	0	0	0	0
4	0	0	0	0
5	1	1	1	1
6	0	0	0	0
7	0	0	0	0
8	1	1	1	1
9	4	4	4	4
10	0	0	0	0
11	2	3	2	1
12	0	0	0	0
13	0	0	0	0
14	2	3	0	0
15	2	3	0	0
16	4	5	2	2
17	4	4	1	1
18	0	0	0	0
19	2	3	2	2
20	2	3	2	2
21	1	1	3	2
22	0	0	0	0
23	0	0	0	0
24	0	.	0	0
25	2	2	0	0
26	0	0	0	0
27	1	1	0	0
28	0	0	0	0
29	4	4	4	4
30	0	0	0	0
31	0	0	0	0
32	4	4	4	4
33	0	0	0	0
34	0	0	2	1
35	2	2	2	2
36	0	0	0	0
37	0	0	0	0
38	4	6	6	4
39	2	2	2	1

	DRdandiL	eyechosen	eyechosenDRdandiS	DRyesorno	DRchosenstage
1	0	1	0	0	0
2	0	1	0	0	0
3	0	1	0	0	0
4	0	1	0	0	0
5	1	1	1	1	1
6	0	1	0	0	0
7	0	1	0	0	0
8	1	1	1	1	1
9	4	1	4	1	2
10	0	1	0	0	0
11	2	1	3	1	1
12	0	1	0	0	0
13	0	1	0	0	0
14	0	1	3	1	1
15	0	1	3	1	1
16	2	1	5	1	2
17	1	1	4	1	2
18	0	1	0	0	0
19	2	1	3	1	1
20	2	1	3	1	1
21	3	2	3	1	1
22	0	1	0	0	0
23	0	1	0	0	0
24	0	2	0	0	0
25	0	1	2	1	1
26	0	1	0	0	0
27	0	1	1	1	1
28	0	1	0	0	0
29	4	1	4	1	2
30	0	1	0	0	0
31	0	1	0	0	0
32	4	1	4	1	2
33	0	1	0	0	0
34	2	2	2	1	1
35	2	1	2	1	1
36	0	1	0	0	0
37	0	1	0	0	0
38	6	1	6	1	2
39	2	1	2	1	1

	eyechosened	VA	IOP	cataract
1	1	.80	15.0000	0
2	1	.60	14.0000	0
3	1	.40	20.0000	0
4	1	.80	18.0000	0
5	1	.15	14.0000	0
6	1	.60	18.0000	2
7	1	1.20	16.0000	0
8	1	.60	18.0000	1
9	1	.80	17.0000	0
10	1	1.00	15.0000	0
11	1	.80	16.0000	0
12	1	.80	.	0
13	1	1.00	18.0000	0
14	1	.40	8.0000	0
15	1	1.00	15.0000	0
16	1	.20	18.0000	2
17	1	.60	17.0000	0
18	1	.30	22.0000	0
19	1	.25	26.0000	0
20	1	1.00	11.0000	0
21	2	.25	17.0000	0
22	1	.80	12.0000	0
23	1	.50	15.0000	0
24	2	.50	18.0000	1
25	1	.10	10.0000	0
26	1	.50	15.0000	0
27	1	.60	15.0000	0
28	1	.60	.	0
29	1	.50	17.0000	0
30	1	.50	15.0000	0
31	1	.60	14.0000	0
32	1	.50	14.0000	0
33	1	.	15.0000	0
34	2	.60	11.0000	1
35	1	.50	21.0000	1
36	1	1.00	18.0000	0
37	1	.80	.	0
38	1	.	12.0000	0
39	1	.25	13.0000	0

renamed_63cd7 12.10 submit.sav

	MA	Hem	HE	CWspot
1	0	0	0	0
2	0	0	0	0
3	0	0	0	0
4	0	0	0	0
5	0	1	0	0
6	0	0	0	0
7	0	0	0	0
8	1	0	0	0
9	0	1	1	0
10	0	0	0	0
11	1	1	1	1
12	0	0	0	0
13	0	0	0	0
14	1	0	0	1
15	0	0	0	1
16	0	0	0	0
17	0	0	0	1
18	0	0	0	0
19	0	0	0	1
20	0	1	0	1
21	0	1	1	1
22	0	0	0	0
23	0	0	0	0
24	0	0	0	0
25	0	0	1	0
26	0	0	0	0
27	0	1	0	0
28	0	0	0	0
29	0	0	0	0
30	0	0	0	0
31	0	0	0	0
32	0	0	0	0
33	0	0	0	0
34	0	0	1	0
35	0	1	1	0
36	0	0	0	0
37	0	0	0	0
38	0	0	0	0
39	0	1	1	0

	VH	TRD	ME	MEandHE
1	0	0	0	0
2	0	0	0	0
3	0	0	0	0
4	0	0	0	0
5	0	0	0	0
6	0	0	0	0
7	0	0	0	0
8	0	0	0	0
9	0	0	0	0
10	0	0	0	0
11	0	0	0	0
12	0	0	0	0
13	0	0	0	0
14	0	0	0	0
15	0	0	0	0
16	1	0	0	0
17	0	0	0	0
18	0	0	0	0
19	0	0	0	0
20	0	0	0	0
21	0	0	1	.
22	0	0	0	0
23	0	0	0	0
24	0	0	0	0
25	0	0	0	0
26	0	0	0	0
27	0	0	0	0
28	0	0	0	0
29	0	0	0	0
30	0	0	0	0
31	0	0	0	0
32	0	0	0	0
33	0	0	0	0
34	0	0	0	0
35	0	0	0	0
36	0	0	0	0
37	0	0	0	0
38	0	1	0	0
39	0	0	1	1

	DRstaged	DRstagei	DRstagedandi	eyedisothers	eyetreothers
1	0	0	0	0	0
2	0	0	0	0	0
3	0	0	0	asteroid hyalosis	0
4	0	0	0	0	0
5	1	1	1	0	0
6	0	0	0	staphyloma	0
7	0	0	0	0	0
8	1	1	1	ERM	0
9	4	4	4	0	PRP
10	0	0	0	0	0
11	3	2	3	0	0
12	0	0	0	0	0
13	0	0	0	0	0
14	3	2	3	0	0
15	3	2	3	0	0
16	5	4	5	0	0
17	4	4	4	0	PRP
18	0	0	0	myopia	0
19	3	2	3	0	0
20	3	2	3	0	0
21	3	2	3	0	0
22	0	0	0	0	0
23	0	0	0	0	0
24	0	0	0	0	0
25	2	2	2	myopia	0
26	0	0	0	0	0
27	1	1	1	0	0
28	0	0	0	ERM	0
29	4	4	4	0	PRP
30	0	0	0	0	0
31	0	0	0	0	0
32	4	4	4	0	PRP
33	0	0	0	0	0
34	2	1	2	0	0
35	2	2	2	0	0
36	0	0	0	0	0
37	0	0	0	0	0
38	6	4	6	0	0
39	2	2	2	0	0

renamed_63cd7 12.10 submit.sav

	imagequality	HRF	HRFstage	HRFyesorno	HRFlocation
1	1	0	0	0	0
2	1	0	0	0	0
3	0	.	.	.	.
4	1	0	0	0	0
5	1	0	0	0	0
6	0	.	.	.	.
7	1	0	0	0	0
8	1	0	0	0	0
9	1	2	2	1	1
10	1	.	.	.	.
11	1	3	2	1	1
12	1	.	.	.	.
13	1	.	.	.	.
14	1	0	0	0	0
15	1	0	0	0	0
16	0	.	.	.	.
17	1	1	1	1	1
18	1	0	0	0	0
19	1	0	0	0	0
20	1	0	0	0	0
21	1	2	2	1	1
22	1	0	0	0	0
23	1	0	0	0	0
24	1	0	0	0	0
25	0	.	.	.	.
26	1	.	.	.	.
27	1	.	.	.	.
28	0	.	.	.	.
29	1	0	0	0	0
30	1	0	0	0	0
31	1	0	0	0	0
32	1	2	2	1	1
33	1	0	0	0	0
34	1	0	0	0	0
35	1	2	2	1	1
36	1	0	0	0	0
37	1	0	0	0	0
38	0	.	.	.	.
39	1	2	2	1	1

	eyeschosen1R2L	VA2	IOP2	cataract2
1	2	.60	14.0000	0
2	2	.80	15.0000	0
3	2	.80	16.0000	0
4	2	.40	18.0000	0
5	2	.40	17.0000	0
6	2	.30	19.0000	2
7	2	1.20	16.0000	0
8	2	.60	18.0000	2
9	2	.60	19.0000	0
10	2	1.00	15.0000	0
11	2	.50	11.0000	0
12	2	.80	18.0000	0
13	2	.05	19.0000	0
14	2	.	9.0000	0
15	2	1.20	15.0000	0
16	2	1.00	19.0000	2
17	2	.25	18.0000	0
18	2	.40	21.0000	0
19	2	.50	21.0000	0
20	2	.80	12.0000	0
21	2	.25	17.0000	0
22	2	.60	12.0000	0
23	2	.15	16.0000	0
24	2	.50	18.0000	1
25	2	.10	15.0000	0
26	2	.60	14.0000	0
27	2	.60	16.0000	0
28	2	.60	.	0
29	2	.50	17.0000	0
30	2	12.00	.0000	0
31	2	.50	14.0000	0
32	2	.20	10.0000	0
33	2	.	15.0000	0
34	2	.60	11.0000	1
35	2	.50	21.0000	1
36	2	1.00	18.0000	0
37	2	.60	.	0
38	2	.	11.0000	0
39	2	1.00	10.0000	0

	MA2	Hem2	HE2	CWspot2	VH2
1	0	0	0	0	0
2	0	0	0	0	0
3	0	0	0	0	0
4	0	0	0	0	0
5	0	1	0	0	0
6	0	0	0	0	0
7	0	0	0	0	0
8	1	0	0	0	0
9	0	1	1	0	0
10	0	0	0	0	0
11	1	1	1	0	0
12	0	0	0	0	0
13	0	0	0	0	0
14	0	0	0	0	0
15	0	0	0	0	0
16	0	1	1	0	0
17	0	1	0	0	0
18	0	0	0	0	0
19	0	0	1	0	0
20	0	1	1	0	0
21	0	1	1	1	0
22	0	0	0	0	0
23	0	0	0	0	0
24	0	0	0	0	0
25	0	0	0	0	0
26	0	0	0	0	0
27	0	0	0	0	0
28	0	0	0	0	0
29	0	0	0	0	1
30	0	0	0	0	0
31	0	0	0	0	0
32	0	0	0	0	0
33	0	0	0	0	0
34	0	0	1	0	0
35	0	0	1	0	0
36	0	0	0	0	0
37	0	0	0	0	0
38	0	0	0	0	0
39	0	1	1	0	0

	TRD2	ME2	MEandHE2	DRstaged2
1	0	0	0	0
2	0	0	0	0
3	0	0	0	0
4	0	0	0	0
5	0	0	0	1
6	0	0	0	0
7	0	0	0	0
8	0	0	0	1
9	0	0	0	4
10	0	0	0	0
11	0	0	0	2
12	0	0	0	0
13	0	0	0	0
14	0	0	0	0
15	0	0	0	0
16	0	0	0	2
17	0	0	0	1
18	0	0	0	0
19	0	0	0	2
20	0	0	0	2
21	0	1	.	3
22	0	0	0	0
23	0	0	0	0
24	0	0	0	0
25	0	0	0	0
26	0	0	0	0
27	0	0	0	0
28	0	0	0	0
29	0	0	0	4
30	0	0	0	0
31	0	0	0	0
32	0	1	1	4
33	0	0	0	0
34	0	0	0	2
35	0	0	0	2
36	0	0	0	0
37	0	0	0	0
38	1	1	0	6
39	0	0	0	2

	DRstagei2	DRstagedandi2	eyedisothers2	eyetreothers2	imagequal[illegible]
1	0	0	0	0	1
2	0	0	0	0	1
3	0	0	0	0	1
4	0	0	0	0	1
5	1	1	0	0	1
6	0	0	0	0	1
7	0	0	0	0	1
8	1	1	0	0	1
9	4	4	0	PRP	1
10	0	0	0	0	1
11	1	2	0	0	1
12	0	0	0	0	1
13	0	0	CRVO ME	injection	0
14	0	0	optic atrophy	0	1
15	0	0	0	0	1
16	2	2	0	0	0
17	1	1	0	0	1
18	0	0	0	0	1
19	2	2	0	0	1
20	2	2	0	0	1
21	2	3	0	0	1
22	0	0	0	0	1
23	0	0	0	0	1
24	0	0	0	0	1
25	0	0	MH	0	0
26	0	0	0	0	1
27	0	0	0	0	1
28	0	0	0	0	1
29	4	4	0	PRP	0
30	0	0	0	0	1
31	0	0	0	0	1
32	4	4	ERM	PRP	1
33	0	0	0	0	1
34	1	2	0	0	1
35	2	2	0	0	1
36	0	0	0	0	1
37	0	0	0	0	1
38	4	6	0	0	0
39	1	2	0	0	1

	HRFstage2	HRFlocation2	filter_$	ln_acr	ln_egfr
1	0	0	0	2.33	4.51
2	0	0	0	3.56	4.08
3	0	0	0	.98	4.59
4	0	0	0	6.04	4.61
5	0	0	0	6.45	4.67
6	0	0	0	.	3.87
7	0	0	0	6.62	4.07
8	0	0	0	6.61	4.49
9	1	1	1	7.33	4.14
10	.	.	0	1.58	4.72
11	3	1	1	5.64	4.43
12	.	.	0	4.59	4.75
13	.	.	0	3.52	4.50
14	.	.	0	4.61	4.59
15	0	0	0	3.66	4.39
16	.	.	0	5.13	4.40
17	0	0	0	6.67	4.09
18	0	0	0	5.43	4.69
19	0	0	0	5.49	4.90
20	2	1	0	4.72	4.34
21	2	1	1	.	4.30
22	0	0	0	6.31	4.37
23	0	0	0	1.70	3.71
24	0	0	0	7.31	3.56
25	.	.	1	6.43	4.02
26	.	.	0	4.04	4.52
27	.	.	0	6.62	4.81
28	0	0	0	5.19	3.83
29	.	.	0	7.70	2.84
30	0	0	0	5.19	4.44
31	0	0	0	6.66	4.77
32	2	1	0	7.49	4.46
33	0	0	0	.	4.45
34	0	0	1	4.60	4.73
35	1	1	1	6.19	4.01
36	0	0	0	3.91	4.75
37	0	0	0	4.09	4.74
38	.	.	0	8.50	3.78
39	1	1	1	5.44	4.25

	egfrgroup4	egfrgroup3	malb2	LogMAR
1	1.00	1.00	1.00	.22
2	3.00	3.00	1.00	.51
3	1.00	1.00	1.00	.92
4	1.00	1.00	2.00	.22
5	1.00	1.00	2.00	1.90
6	3.00	3.00	.	.51
7	3.00	3.00	2.00	-.18
8	2.00	2.00	2.00	.51
9	2.00	2.00	2.00	.22
10	1.00	1.00	1.00	.00
11	2.00	2.00	1.00	.22
12	1.00	1.00	1.00	.22
13	2.00	2.00	1.00	.00
14	1.00	1.00	1.00	.92
15	2.00	2.00	1.00	.00
16	2.00	2.00	1.00	1.61
17	3.00	3.00	2.00	.51
18	1.00	1.00	1.00	1.20
19	1.00	1.00	1.00	1.39
20	2.00	2.00	1.00	.00
21	2.00	2.00	.	1.39
22	2.00	2.00	2.00	.22
23	3.00	3.00	1.00	.69
24	3.00	3.00	2.00	.69
25	3.00	3.00	2.00	2.30
26	1.00	1.00	1.00	.69
27	1.00	1.00	2.00	.51
28	3.00	3.00	1.00	.51
29	4.00	3.00	2.00	.69
30	2.00	2.00	1.00	.69
31	1.00	1.00	2.00	.51
32	2.00	2.00	2.00	.69
33	2.00	2.00	.	.
34	1.00	1.00	1.00	.51
35	3.00	3.00	2.00	.69
36	1.00	1.00	1.00	.00
37	1.00	1.00	1.00	.22
38	3.00	3.00	2.00	.
39	2.00	2.00	1.00	1.39

	No	ID	Sex	Age	HBPstage	HBP	HBPyears
40	40	2274918	1	61	3	1	10.0
41	41	2275052	1	61	2	1	6.0
42	42	2275429	1	57	0	0	.0
43	43	2275579	1	46	2	1	.1
44	44	2277712	1	61	2	1	.
45	45	1230320	2	80	1	1	22.0
46	46	1321372	1	58	.	.	.
47	47	1333622	2	46	0	0	.0
48	48	1407393	1	62	1	1	10.0
49	49	1737969	2	62	1	1	10.0
50	50	12118	1	69	1	1	5.0
51	51	1310920	1	79	1	1	30.0
52	52	1336743	1	68	2	1	13.0
53	53	1348499	1	48	0	0	.0
54	54	2281548	1	53	0	0	.0
55	55	2281804	1	54	1	1	.
56	56	2281969	2	74	3	1	2.0
57	57	2283607	2	64	3	1	31.0
58	58	2284466	1	52	0	0	.0
59	59	497572	1	66	0	0	.0
60	60	1209566	2	66	3	1	34.0
61	61	1325998	2	54	1	1	2.0
62	62	1445480	1	60	0	0	.0
63	63	1507410	1	48	2	1	1.0
64	64	2284689	2	86	1	1	40.0
65	65	2286682	2	77	3	1	10.0
66	66	205349	1	50	2	1	.
67	67	1349290	1	47	1	1	.5
68	68	1440324	1	70	1	1	.
69	69	1628369	1	61	1	1	4.0
70	70	2288168	2	78	3	1	8.0
71	71	2289578	1	45	0	0	.0
72	72	2290664	1	55	3	1	25.0
73	73	2292080	2	71	3	1	.
74	74	1058353	2	75	3	1	30.0
75	75	1309348	1	53	2	1	15.0
76	76	2012244	1	81	3	1	30.0
77	77	2215869	1	56	1	1	1.0
78	78	2216578	1	37	0	0	.0

	CHD	PA	PN	UA	HCY	DNstage	DNstageadjust
40	0	0	1	0	0	1	3
41	0	1	0	0	0	4	4
42	1	1	0	0	1	1	3
43	0	0	1	0	0	2	3
44	0	1	1	0	0	3	4
45	0	1	0	0	0	3	2
46	.	.	.	.	.	.	.
47	0	1	0	0	0	2	.
48	0	1	0	0	0	0	4
49	0	1	0	1	0	3	4
50	0	0	1	0	1	.	3
51	0	0	1	1	0	4	4
52	1	1	1	0	0	3	2
53	0	1	0	0	0	1	1
54	0	1	1	0	0	3	3
55	0	1	0	0	0	4	4
56	0	0	1	0	0	3	4
57	0	0	0	0	0	3	4
58	0	0	1	0	0	1	3
59	0	1	0	0	0	3	3
60	1	0	1	0	0	3	4
61	1	1	0	1	0	2	3
62	1	1	.	0	0	4	4
63	0	0	0	0	0	4	4
64	0	0	1	0	0	3	3
65	0	0	1	1	1	4	4
66	0	1	0	0	0	4	.
67	0	0	0	0	0	4	5
68	0	1	0	0	0	4	4
69	1	1	0	0	0	1	2
70	0	1	1	0	0	2	3
71	0	1	0	1	0	3	3
72	0	0	0	0	0	5	3
73	0	0	0	0	0	3	3
74	0	1	1	0	0	4	3
75	0	1	0	0	0	1	1
76	0	1	0	1	0	3	3
77	0	1	1	1	0	4	4
78	0	0	1	0	0	1	3

	DNstage2	DNstage3	KDeGFR	UPstage	UPstage2	HL
40	1	2	1	2	1	1
41	2	3	2	3	2	1
42	1	2	1	2	1	1
43	1	2	3	.	.	1
44	2	3	1	3	2	0
45	1	1	2	1	1	1
46	.	.	.	.	.	.
47	.	.	1	1	1	1
48	2	3	3	3	2	1
49	2	3	3	3	2	0
50	1	2	3	2	1	1
51	2	3	2	3	2	1
52	1	1	2	1	1	0
53	1	1	1	1	1	1
54	1	2	2	2	1	0
55	2	3	1	3	2	0
56	2	3	3	3	2	0
57	2	3	3	3	2	0
58	1	2	1	2	1	1
59	1	2	2	2	1	1
60	2	3	3	3	2	1
61	1	2	1	2	1	1
62	2	3	2	3	2	0
63	2	3	3	3	2	1
64	1	2	2	2	1	1
65	2	3	2	3	2	1
66	.	.	2	.	.	1
67	2	3	5	.	.	1
68	2	3	3	3	2	1
69	1	1	2	.	.	1
70	1	2	2	2	1	1
71	1	2	1	2	1	1
72	1	2	2	2	1	0
73	1	2	1	2	1	1
74	1	2	2	2	1	0
75	1	1	1	.	.	1
76	1	2	2	2	1	1
77	2	3	2	3	2	1
78	1	2	1	2	1	1

	HLyears	HLmedicine	DMtype	DMyears
40	.25	1	2	12.0
41	6.00	1	2	6.0
42	.00	0	2	8.0
43	.10	1	2	8.0
44	.00	0	2	20.0
45	9.00	1	2	16.0
46	.	.	.	.
47	.00	0	2	12.0
48	10.00	1	2	14.0
49	.00	0	2	12.0
50	28.00	1	2	36.0
51	.00	0	2	30.0
52	.00	0	2	20.0
53	17.00	1	2	8.0
54	.00	0	2	14.0
55	.00	0	2	10.0
56	.00	0	2	.4
57	.00	0	2	16.0
58	.00	0	1	15.0
59	.	1	1	35.0
60	5.00	1	2	36.0
61	.	1	2	14.0
62	.00	0	2	20.0
63	1.00	1	2	13.0
64	40.00	1	2	40.0
65	10.00	1	2	10.0
66	6.00	0	2	8.0
67	3.00	1	2	20.0
68	.00	0	2	29.0
69	.00	0	2	20.0
70	8.00	0	2	8.0
71	.	1	2	12.0
72	.00	0	2	6.0
73	.00	0	2	5.0
74	.00	0	2	20.0
75	.00	0	2	8.0
76	30.00	1	2	30.0
77	1.00	1	2	20.0
78	1.00	1	2	.

...	HbA1c	Height	WeightKg	BMI
40	8.00	158	61.0000	24.4000
41	8.30	179	92.0000	28.7000
42	.	170	54.0000	18.6800
43	7.50	182	83.0000	25.0000
44	8.30	170	80.0000	27.6800
45	6.50	158	67.0000	26.8200
46	.	.	.	.
47	7.10	150	60.0000	26.7000
48	8.40	175	77.0000	25.1400
49	.	160	73.0000	28.5200
50	8.40	178	77.9000	24.5800
51	7.70	161	74.6000	28.6000
52	.	180	83.0000	24.5200
53	6.10	171	91.0000	31.4800
54	9.80	180	71.0000	21.9100
55	8.90	170	70.0000	24.2200
56	15.30	153	56.0000	23.9000
57	5.00	156	56.5000	23.2000
58	13.60	178	62.5000	19.7000
59	11.80	160	61.0000	23.8300
60	12.10	162	29.4500	30.2700
61	9.70	168	87.6000	31.0000
62	7.70	163	64.3500	24.6100
63	9.80	177	88.0000	28.1000
64	8.70	159	51.4000	20.3300
65	7.70	159	59.0000	23.3400
66	10.60	177	96.7000	31.0000
67	.	175	90.0000	29.3900
68	10.40	165	65.0000	23.8700
69	.	160	63.0000	24.6000
70	11.00	160	52.0000	21.6000
71	10.60	175	75.0000	25.9500
72	7.50	170	110.0000	38.0600
73	7.30	160	83.7000	32.7000
74	9.80	160	72.0000	28.1300
75	7.70	170	74.2000	25.1000
76	6.80	170	68.0000	23.5300
77	6.20	176	72.0000	23.2400
78	9.00	185	92.9000	27.3000

	AC	SBP	DBP	SCRumolL
40	93.5	198	82	69.90
41	102.0	113	69	83.30
42	77.5	109	71	66.30
43	95.5	142	77	128.30
44	.	151	85	73.70
45	.	143	76	61.60
46	.	.	.	.
47	.	122	85	52.30
48	90.0	148	85	155.20
49	.	180	98	98.00
50	100.0	129	52	111.80
51	107.0	151	68	102.00
52	94.0	129	77	86.00
53	.	110	74	72.30
54	.	126	80	73.40
55	.	128	66	62.70
56	88.5	160	71	92.70
57	75.0	136	80	143.90
58	82.0	96	63	66.60
59	.	141	91	89.90
60	113.0	170	70	119.50
61	105.0	126	72	53.80
62	.	123	57	101.60
63	103.0	94	65	125.50
64	82.0	146	55	63.10
65	.	164	69	81.20
66	90.0	168	88	101.50
67	.	113	67	705.60
68	102.0	133	76	155.20
69	63.0	104	63	105.50
70	82.0	120	80	49.00
71	.	133	87	68.50
72	120.0	181	111	113.20
73	102.5	139	66	40.10
74	.	138	77	64.80
75	89.0	148	80	56.10
76	85.0	179	66	115.30
77	.	157	90	87.20
78	101.0	124	72	51.20

	eGFRmlmin1.73m2	CKDstage	DKDstage	UPstageadj	UAadj
40	97.01	1	3	2	413.00
41	87.25	2	4	3	497.00
42	101.97	1	3	2	357.00
43	57.51	3	3	.	282.00
44	94.93	1	4	3	361.00
45	82.04	2	2	1	216.00
46	.	.	.	.	.
47	109.94	1	.	1	363.00
48	40.83	3	4	3	390.00
49	53.48	3	4	3	482.00
50	55.68	3	3	2	303.00
51	60.19	2	4	3	467.00
52	79.92	2	2	1	523.00
53	104.83	1	1	1	416.00
54	89.22	2	3	2	360.00
55	106.56	1	4	3	387.00
56	52.57	3	4	3	316.00
57	33.38	3	4	3	312.00
58	105.42	1	3	2	362.00
59	76.82	2	3	2	238.00
60	40.91	3	4	3	408.00
61	100.34	1	3	2	422.00
62	69.11	2	4	3	328.00
63	58.24	3	4	3	488.00
64	76.94	2	3	2	287.00
65	60.42	2	4	3	497.00
66	74.23	2	.	.	426.00
67	7.27	5	5	.	376.00
68	38.60	3	4	3	468.00
69	65.57	2	2	.	370.00
70	89.71	2	3	2	276.00
71	109.46	1	3	2	554.00
72	62.81	2	3	2	250.00
73	100.65	1	3	2	343.00
74	85.16	2	3	2	295.00
75	111.92	1	1	.	139.00
76	61.01	2	3	2	211.00
77	85.51	2	4	3	445.00
78	131.14	1	3	2	384.00

	ACRmgmmol	ACRmgg	UP24hg	CHOLmmolL	TGmmolL
40	24.22	214.10	.	3.97	.69
41	35.62	314.88	.67	3.67	4.99
42	4.80	42.43	.02	5.16	1.15
43	.	.	.	2.83	2.04
44	63.58	562.05	.70	5.32	.70
45	.48	4.24	.04	3.88	.99
46	.	.	.	.	.
47	.93	8.22	.	4.98	1.03
48	41.54	367.21	.80	5.08	2.89
49	1031.47	9118.19	2.60	2.80	2.49
50	5.48	48.44	.04	2.73	1.38
51	60.75	537.03	.97	5.11	1.70
52	1.37	12.11	.	2.46	2.03
53	.44	3.89	.	4.37	1.98
54	21.40	189.18	.23	4.43	2.25
55	773.47	6837.47	5.31	3.85	2.64
56	407.18	3599.47	3.04	5.44	1.34
57	270.01	2386.89	1.27	1.65	3.76
58	9.37	82.83	.18	3.17	1.10
59	28.55	252.38	.34	3.37	2.21
60	74.44	658.05	.49	3.03	1.84
61	6.30	55.69	.16	2.68	2.01
62	71.40	631.18	1.42	4.27	1.69
63	183.84	1625.15	2.58	3.95	3.58
64	21.92	193.77	.	4.74	3.51
65	209.36	1850.74	.	4.94	2.96
66	.	.	8.26	5.32	10.90
67	.	.	.	4.19	3.22
68	116.14	1026.68	1.53	5.34	2.66
69	.	.	.	5.80	3.10
70	7.06	62.41	.	7.20	2.62
71	19.97	176.53	.	7.96	22.36
72	14.27	126.15	3.50	3.57	1.56
73	12.92	114.21	.11	5.65	2.18
74	27.14	239.92	.18	3.64	.84
75	.	.	.22	3.55	1.35
76	22.16	195.89	.31	3.19	1.19
77	89.68	792.77	.	2.43	.78
78	3.98	35.18	.	4.22	3.08

	HDLCmmolL	LDLCmmolL	HCYumolL	GLUmmolL
40	1.20	3.37	14.54	5.78
41	.56	2.22	12.50	8.19
42	.98	3.49	13.07	8.59
43	.74	1.70	22.54	5.40
44	.96	3.55	17.33	12.80
45	1.06	2.58	11.79	4.89
46	.	.	.	.
47	1.48	2.97	8.37	6.30
48	.87	3.23	15.82	11.49
49	.89	1.55	13.15	.
50	.97	.43	11.39	4.26
51	1.01	3.39	12.04	7.95
52	1.07	1.20	15.19	.
53	1.01	2.70	11.59	.
54	.93	2.94	16.77	.
55	.96	3.79	11.56	.
56	1.29	3.50	15.19	8.13
57	.73	2.71	12.34	5.54
58	.89	2.12	13.44	.
59	.86	1.86	12.87	.
60	1.01	1.81	14.55	11.17
61	.99	1.61	12.02	13.86
62	.40	2.55	13.17	5.86
63	.78	2.46	15.17	.
64	1.04	2.94	13.67	10.16
65	1.14	3.19	16.22	9.22
66	.79	2.33	14.30	7.25
67	.64	2.88	.	7.59
68	1.05	3.36	.	10.11
69	5.24	3.54	14.70	5.62
70	1.30	4.77	11.42	6.93
71	1.52	1.86	12.02	7.69
72	1.16	2.03	17.51	13.21
73	1.11	3.68	10.09	6.39
74	1.34	2.15	14.38	7.73
75	1.07	2.10	13.45	7.83
76	.66	1.97	15.20	9.28
77	.97	1.31	15.65	7.82
78	.94	2.48	10.60	6.18

	INSuIUml	CPngml	@0.5hGLU	@0.5hINS
40	12.81	.86	7.98	17.02
41	12.55	4.42	11.60	15.32
42	12.48	.64	10.84	12.19
43	5.24	1.29	6.52	5.92
44	.	.	.	.
45	9.84	1.29	.	.
46	.	.	.	.
47	23.57	1.28	13.96	57.04
48	20.06	5.35	12.26	32.66
49	6.08	1.68	.	.
50	8.77	3.01	8.80	.
51	11.31	1.75	11.97	23.03
52	.	.	.	.
53	.	.	.	.
54	.	.	.	.
55	.	.	13.59	7.45
56	13.59	1.84	7.95	13.00
57	8.04	2.19	8.64	10.99
58	15.09	1.05	.	.
59	.	.	.	.
60	13.16	70.03	.	.
61	13.90	3.44	15.37	18.46
62	5.97	1.50	12.44	11.81
63	.	.	.	.
64	71.49	1.76	17.09	73.44
65	12.42	2.62	.	.
66	4.97	1.66	8.48	13.38
67	.	.	.	.
68	15.02	.59	11.46	14.39
69	6.08	.49	8.77	6.71
70	11.48	2.87	8.65	15.86
71	15.19	3.49	9.86	20.50
72	.	.	.	.
73	15.85	2.76	7.25	16.65
74	19.59	2.37	.	.
75	9.27	1.30	13.20	18.16
76	16.01	4.45	9.34	18.02
77	9.37	2.26	.	.
78	1.32	2.68	6.33	19.38

	@0.5hCP	@1hGLU	@1hINS	@1hCP
40	1.23	13.82	25.53	1.93
41	4.29	17.80	35.91	6.21
42	.66	14.89	15.12	.69
43	1.60	8.21	6.91	1.82
44	.	.	.	.
45	.	13.46	28.80	3.56
46	.	.	.	.
47	2.45	16.64	72.71	4.49
48	6.01	13.64	32.03	6.38
49	.	.	.	.
50	2.50	12.82	27.37	3.59
51	2.36	15.18	36.09	3.17
52	.	.	.	.
53	.	.	.	.
54	.	.	.	.
55	3.00	17.25	11.90	3.58
56	1.76	8.64	12.49	1.82
57	2.50	12.58	15.01	2.90
58	.	.	.	.
59	.	.	.	.
60	.	.	.	.
61	3.42	16.74	19.33	3.87
62	1.70	16.55	29.80	3.27
63	.	.	.	.
64	2.29	22.72	105.50	3.46
65	.	18.07	30.84	4.39
66	2.22	10.81	15.24	2.49
67	.	.	.	.
68	.60	13.87	14.50	.60
69	1.25	13.11	11.31	1.87
70	3.00	9.55	18.68	3.34
71	4.14	12.70	23.77	4.56
72	.	.	.	.
73	2.81	9.42	31.42	3.28
74	.	13.03	27.45	2.80
75	1.61	18.10	30.17	2.34
76	4.04	12.32	28.21	4.29
77	.	6.82	9.25	2.04
78	2.57	7.84	23.26	2.89

	@2hGLU	@2hINS	@2hCP	DRdosmesticR
40	17.77	30.02	3.08	2
41	16.85	33.29	8.75	0
42	22.67	15.14	.93	3
43	12.31	10.11	2.91	5
44	.	.	.	3
45	14.55	31.28	5.30	0
46	.	.	.	6
47	16.32	65.31	4.60	4
48	19.91	57.70	8.20	0
49	11.07	16.66	4.28	0
50	17.79	22.17	4.74	0
51	20.87	63.80	5.11	.
52	.	.	.	0
53	.	.	.	0
54	.	.	.	2
55	22.20	7.33	3.22	2
56	.	14.67	2.06	5
57	10.59	30.75	4.50	.
58	.	44.63	2.40	0
59	.	.	.	6
60	14.29	2.90	3.15	4
61	19.34	26.30	4.72	3
62	19.33	37.07	4.75	2
63	.	.	.	2
64	22.08	100.90	4.28	0
65	23.70	50.22	6.79	2
66	16.38	21.82	3.62	3
67	.	.	.	6
68	20.39	23.46	.84	5
69	21.84	15.21	3.58	6
70	11.00	27.28	5.36	1
71	11.86	25.56	5.36	3
72	.	.	.	6
73	12.36	38.01	4.03	2
74	16.56	38.52	3.91	0
75	16.20	10.58	3.00	1
76	16.14	36.52	6.06	5
77	4.39	5.51	1.55	3
78	11.27	39.33	4.71	0

	DRinternatioinalR	DRdandiR	DRdosmesticL	DRinternatioinalL
40	2	2	2	2
41	0	0	0	0
42	2	3	0	0
43	4	5	3	3
44	2	3	0	0
45	0	0	.	.
46	4	6	6	4
47	4	4	4	4
48	0	0	0	0
49	0	0	2	2
50	0	0	0	0
51	0	.	0	0
52	0	0	0	0
53	0	0	0	0
54	1	2	2	1
55	2	2	2	2
56	4	4	5	4
57	.	.	.	.
58	0	0	3	2
59	4	6	6	4
60	3	4	4	3
61	2	3	2	2
62	2	2	0	0
63	2	2	2	2
64	0	0	0	0
65	2	2	0	0
66	2	3	3	2
67	4	6	.	.
68	4	4	5	4
69	4	6	2	1
70	1	1	0	0
71	2	3	2	1
72	4	6	6	4
73	1	2	2	1
74	0	0	0	0
75	1	1	0	0
76	4	4	5	4
77	2	3	1	1
78	0	0	0	0

	DRdandiL	eyechosen	eyechosenD[illegible]	DRyesorno	DRchosensta[illegible]
40	2	1	2	1	1
41	0	1	0	0	0
42	0	1	3	1	1
43	3	1	5	1	2
44	0	1	3	1	1
45	.	1	0	0	0
46	6	1	6	1	2
47	4	1	4	1	2
48	0	1	0	0	0
49	2	2	2	1	1
50	0	1	0	0	0
51	0	2	0	0	0
52	0	1	0	0	0
53	0	1	0	0	0
54	2	1	2	1	1
55	2	1	2	1	1
56	4	1	4	1	2
57	.	.	.	.	.
58	3	2	3	1	1
59	6	1	6	1	2
60	4	1	4	1	2
61	2	1	3	1	1
62	0	1	2	1	1
63	2	1	2	1	1
64	0	1	0	0	0
65	0	1	2	1	1
66	3	1	3	1	1
67	.	1	6	1	2
68	4	1	4	1	2
69	2	1	6	1	2
70	0	1	1	1	1
71	2	1	3	1	1
72	6	1	6	1	2
73	2	1	2	1	1
74	0	1	0	0	0
75	0	1	1	1	1
76	4	1	4	1	2
77	1	1	3	1	1
78	0	1	0	0	0

	eyechosened	VA	IOP	cataract
40	1	.40	12.0000	0
41	1	.80	13.0000	0
42	1	.60	12.0000	0
43	1	.	11.0000	1
44	1	.80	10.0000	0
45	1	.50	.	1
46	1	.	14.0000	1
47	1	.50	23.0000	1
48	1	.50	14.0000	0
49	2	.50	15.0000	0
50	1	1.00	8.8000	0
51	2	.60	12.0000	1
52	1	.60	9.0000	1
53	1	1.20	17.0000	0
54	1	.	.	1
55	1	.40	13.0000	1
56	1	.25	.	3
57	1	.30	19.0000	3
58	2	.40	14.0000	0
59	1	.25	16.0000	3
60	1	.10	12.0000	0
61	1	1.00	13.0000	0
62	1	.80	12.0000	0
63	1	.30	17.0000	0
64	1	.40	15.0000	0
65	1	.40	8.0000	2
66	1	1.00	14.0000	1
67	1	.	.	2
68	1	.50	17.0000	2
69	1	.50	14.0000	1
70	1	.40	11.0000	1
71	1	.40	18.0000	1
72	1	.	10.0000	1
73	1	.25	12.0000	1
74	1	.80	19.0000	0
75	1	.40	11.0000	0
76	1	.50	.	2
77	1	.80	.	1
78	1	.80	20.0000	0

	MA	Hem	HE	CWspot
40	0	1	1	0
41	0	0	0	0
42	0	1	0	1
43	0	0	0	0
44	0	1	0	1
45	0	0	0	0
46	0	0	0	0
47	0	0	0	0
48	0	0	0	0
49	1	1	1	0
50	0	0	0	0
51	0	0	0	0
52	0	0	0	0
53	0	0	0	0
54	0	0	1	0
55	0	1	1	0
56	0	0	1	0
57	.	.	.	.
58	0	0	0	1
59	0	0	0	0
60	0	0	0	0
61	0	1	0	1
62	0	1	1	0
63	0	1	1	0
64	0	0	0	0
65	0	1	1	0
66	0	1	1	1
67	.	.	.	.
68	0	0	0	0
69	0	0	0	0
70	1	0	0	0
71	0	1	1	1
72	0	0	0	0
73	0	0	1	0
74	0	.	0	0
75	0	1	0	0
76	0	0	0	0
77	0	1	0	1
78	0	0	0	0

	VH	TRD	ME	MEandHE
40	0	0	1	1
41	0	0	0	0
42	0	0	0	0
43	1	0	1	0
44	0	0	0	0
45	0	0	0	0
46	1	1	0	0
47	1	0	0	0
48	0	0	0	0
49	0	0	0	0
50	0	0	0	0
51	0	0	0	0
52	0	0	0	0
53	0	0	0	0
54	0	0	0	0
55	0	0	1	1
56	0	0	0	0
57	.	.	.	.
58	0	0	0	0
59	0	0	0	0
60	0	0	0	0
61	0	0	0	0
62	0	0	0	0
63	0	0	0	0
64	0	0	0	0
65	0	0	1	1
66	0	0	0	0
67	.	.	.	.
68	0	0	0	0
69	0	1	0	0
70	0	0	0	0
71	0	0	0	0
72	0	1	0	0
73	0	0	0	0
74	0	0	0	0
75	0	0	0	0
76	0	0	0	0
77	0	0	0	0
78	0	0	0	0

	DRstaged	DRstagei	DRstagedandi	eyedisothers	eyetreothers
40	2	2	2	0	0
41	0	0	0	0	0
42	3	2	3	0	0
43	5	4	5	0	injecton
44	3	2	3	0	0
45	0	0	0	0	0
46	6	4	6	0	0
47	4	4	4	0	PPV
48	0	0	0	0	0
49	2	2	2	0	0
50	0	0	0	0	0
51	0	0	0	0	0
52	0	0	0	0	0
53	0	0	0	0	0
54	2	1	2	0	0
55	2	2	2	0	0
56	5	4	4	0	PPVandPa...
57	.	.	.	asteroid hyalosis	0
58	3	2	3	0	0
59	6	4	6	0	PPV
60	4	3	4	0	PRP
61	3	2	3	0	0
62	2	2	2	0	0
63	2	2	2	0	0
64	0	0	0	PED	0
65	2	2	2	ERM	0
66	3	2	3	BRVO	0
67	6	4	6	0	PPVandSO
68	5	4	4	ERM	PPV
69	6	4	6	0	0
70	1	1	1	staphyloma	0
71	3	2	3	0	0
72	6	4	6	0	0
73	2	1	2	staphyloma	0
74	0	0	0	0	0
75	1	1	1	0	0
76	5	4	4	ERM	PPV
77	3	2	3	0	0
78	0	0	0	0	0

	imagequality	HRF	HRFstage	HRFyesorno	HRFlocation
40	1	1	1	1	1
41	1	0	0	0	0
42	0	.	.	.	.
43	0	1	1	1	1
44	1	0	0	0	0
45	1	0	0	0	0
46	0	.	.	.	.
47	1	.	.	.	.
48	1	0	0	0	0
49	1	1	1	1	1
50	1	0	0	0	0
51	1	0	0	0	0
52	1	0	0	0	0
53	1	0	0	0	0
54	1	1	1	1	1
55	1	2	2	1	1
56	1	2	2	1	1
57	0	.	.	.	.
58	1	0	0	0	0
59	1	.	.	.	.
60	0	.	.	.	.
61	1	0	0	0	0
62	1	1	1	1	1
63	1	1	1	1	1
64	1	.	.	.	.
65	1	2	2	1	1
66	1	0	0	0	0
67	0	.	.	.	.
68	1	1	1	1	1
69	0	.	.	.	.
70	1	0	0	0	0
71	1	2	2	1	1
72	0	.	.	.	.
73	0	.	.	.	.
74	1	0	0	0	0
75	1	0	0	0	0
76	0	.	.	.	.
77	1	0	0	0	0
78	1	0	0	0	0

	eyeschosen1R2L	VA2	IOP2	cataract2
40	2	.50	10.0000	0
41	2	1.00	10.0000	0
42	2	.60	11.0000	0
43	2	.10	12.0000	1
44	2	.80	13.0000	0
45	2	.	.	1
46	2	.	22.0000	1
47	2	.80	21.0000	1
48	2	.80	15.0000	0
49	2	.50	15.0000	0
50	2	1.00	9.3000	0
51	2	.60	12.0000	1
52	2	.60	10.0000	1
53	2	1.20	22.0000	0
54	2	.	.	1
55	2	.20	11.0000	1
56	2	.40	.	3
57	2	.10	16.0000	3
58	2	.40	14.0000	0
59	2	.30	14.0000	1
60	2	.10	12.0000	0
61	2	1.00	17.0000	0
62	2	.80	10.0000	0
63	2	.60	17.0000	0
64	2	.06	15.0000	0
65	2	.30	8.0000	2
66	2	.80	16.0000	1
67	2	.	.	1
68	2	.25	20.0000	2
69	2	.50	15.0000	1
70	2	.50	12.0000	1
71	2	.50	17.0000	1
72	2	.	10.0000	1
73	2	.40	12.0000	1
74	2	.60	17.0000	0
75	2	.80	11.0000	0
76	2	.40	.	2
77	2	1.00	.	1
78	2	1.00	20.0000	0

	MA2	Hem2	HE2	CWspot2	VH2
40	0	1	1	0	0
41	0	0	0	0	0
42	0	0	0	0	0
43	0	1	0	1	0
44	0	0	0	0	0
45	.	.	.	.	.
46	0	0	0	0	1
47	0	0	0	0	1
48	0	0	0	0	0
49	1	1	1	0	0
50	0	0	0	0	0
51	0	0	0	0	0
52	0	0	0	0	0
53	0	0	0	0	0
54	0	1	1	0	0
55	0	1	1	0	0
56	0	1	1	0	0
57	.	.	.	.	.
58	0	0	0	1	0
59	0	0	0	0	1
60	0	0	0	0	0
61	0	1	1	0	0
62	0	0	0	0	0
63	0	1	1	0	0
64	0	0	0	0	0
65	0	0	0	0	0
66	0	1	1	1	0
67	.	.	.	.	.
68	0	0	0	0	0
69	0	1	0	0	0
70	0	0	0	0	0
71	0	1	1	0	0
72	0	0	0	0	0
73	0	1	1	0	0
74	0	0	0	0	0
75	0	0	0	0	0
76	0	0	0	0	0
77	0	1	0	0	0
78	0	0	0	0	0

	TRD2	ME2	MEandHE2	DRstaged2
40	0	1	1	2
41	0	0	0	0
42	0	0	0	0
43	0	1	0	3
44	0	0	0	0
45	.	.	.	.
46	1	0	0	6
47	0	0	0	4
48	0	0	0	0
49	0	0	0	2
50	0	0	0	0
51	0	0	0	0
52	0	0	0	0
53	0	0	0	0
54	0	0	0	2
55	0	1	1	2
56	0	0	0	5
57	.	.	.	.
58	0	0	0	3
59	1	0	0	6
60	0	0	0	4
61	0	0	?	?
62	0	0	?	?
63	0	0	?	?
64	0	0	?	?
65	0	0	?	?
66	0	0	?	?
67	.	.	?	?
68	0	0	?	?
69	0	0	?	?
70	0	0	?	?
71	0	0	?	?
72	1	0	?	?
73	0	0	?	?
74	0	0	?	?
75	0	0	0	0
76	0	0	0	5
77	0	0	0	1
78	0	0	0	0

	DRstagei2	DRstagedandi2	eyedisothers2	eyetreothers2	imagequal
40	2	2	0	0	1
41	0	0	0	0	1
42	0	0	0	0	0
43	3	3	0	injectiobn	1
44	0	0	0	0	1
45	.	.	myopia		0
46	4	6	0	PPV	0
47	4	4	0	PPV	1
48	0	0	0	0	1
49	2	2	0	0	1
50	0	0	0	0	1
51	0	0	0	0	1
52	0	0	0	0	1
53	0	0	0	0	1
54	1	2	0	0	1
55	2	2	0	0	1
56	4	4	0	PPVPI	1
57	.	.	astro	0	0
58	2	3	0	0	1
59	4	6	0	0	0
60	3	4	0	PRP	0
61	2	2	0	0	1
62	0	0	0	0	1
63	2	2	0	0	1
64	0	0	PED	0	1
65	0	0	0	0	1
66	2	3	0	0	1
67	.	.			0
68	4	4	ERM	PRP	1
69	1	2	0	0	1
70	0	0	ERM	0	1
71	1	2	0	0	1
72	4	6	0	PRP	0
73	1	2	0	0	1
74	0	0	0	0	1
75	0	0	0	0	1
76	4	4	ERM	PPV	0
77	1	1	0	0	1
78	0	0	0	0	1

	HRFstage2	HRFlocation2	filter_$	ln_acr	ln_egfr
40	1	1	1	5.37	4.57
41	0	0	0	5.75	4.47
42	.	.	0	3.75	4.62
43	1	1	0	.	4.05
44	0	0	0	6.33	4.55
45	.	.	0	1.45	4.41
46	.	.	0	.	.
47	.	.	0	2.11	4.70
48	0	0	0	5.91	3.71
49	1	1	1	9.12	3.98
50	0	0	0	3.88	4.02
51	0	0	0	6.29	4.10
52	0	0	0	2.49	4.38
53	0	0	0	1.36	4.65
54	1	3	1	5.24	4.49
55	1	1	1	8.83	4.67
56	1	1	1	8.19	3.96
57	.	.	.	7.78	3.51
58	0	0	0	4.42	4.66
59	.	.	0	5.53	4.34
60	.	.	0	6.49	3.71
61	0	0	0	4.02	4.61
62	0	0	1	6.45	4.24
63	1	1	1	7.39	4.06
64	.	.	0	5.27	4.34
65	0	0	1	7.52	4.10
66	1	1	1	.	4.31
67	.	.	.	.	1.98
68	1	1	0	6.93	3.65
69	0	0	0	.	4.18
70	0	0	0	4.13	4.50
71	1	1	1	5.17	4.70
72	.	.	0	4.84	4.14
73	0	0	1	4.74	4.61
74	0	0	0	5.48	4.44
75	0	0	0	.	4.72
76	.	.	0	5.28	4.11
77	0	0	0	6.68	4.45
78	0	0	0	3.56	4.88

	egfrgroup4	egfrgroup3	malb2	LogMAR
40	1.00	1.00	1.00	.92
41	2.00	2.00	2.00	.22
42	1.00	1.00	1.00	.51
43	3.00	3.00	.	.
44	1.00	1.00	2.00	.22
45	2.00	2.00	1.00	.69
46	.	.	.	.
47	1.00	1.00	1.00	.69
48	3.00	3.00	2.00	.69
49	3.00	3.00	2.00	.69
50	3.00	3.00	1.00	.00
51	2.00	2.00	2.00	.51
52	2.00	2.00	1.00	.51
53	1.00	1.00	1.00	-.18
54	2.00	2.00	1.00	.
55	1.00	1.00	2.00	.92
56	3.00	3.00	2.00	1.39
57	3.00	3.00	2.00	1.20
58	1.00	1.00	1.00	.92
59	2.00	2.00	1.00	1.39
60	3.00	3.00	2.00	2.30
61	1.00	1.00	1.00	.00
62	2.00	2.00	2.00	.22
63	3.00	3.00	2.00	1.20
64	2.00	2.00	1.00	.92
65	2.00	2.00	2.00	.92
66	2.00	2.00	.	.00
67	4.00	3.00	.	.
68	3.00	3.00	2.00	.69
69	2.00	2.00	.	.69
70	2.00	2.00	1.00	.92
71	1.00	1.00	1.00	.92
72	2.00	2.00	1.00	.
73	1.00	1.00	1.00	1.39
74	2.00	2.00	1.00	.22
75	1.00	1.00	.	.92
76	2.00	2.00	1.00	.69
77	2.00	2.00	2.00	.22
78	1.00	1.00	1.00	.22

	No	ID	Sex	Age	HBPstage	HBP	HBPyears
79	79	2255444	1	65	0	0	.0
80	80	2292144	2	65	3	1	5.0
81	81	2294433	1	64	3	1	5.0
82	82	541223	2	55	1	1	5.0
83	83	2298168	2	53	3	1	5.0
84	84	1036220	2	76	3	1	17.0
85	85	1165337	1	86	3	1	10.0
86	86	1185337	2	66	1	1	8.0
87	87	1238394	2	65	1	1	.5
88	88	1390223	1	40	0	0	.0
89	89	1414470	1	68	0	0	.0
90	90	1446120	2	72	1	1	3.0
91	91	1487153	2	62	3	1	20.0
92	92	1501823	1	62	3	1	1.0
93	93	2004418	1	48	3	1	6.0
94	94	2021635	1	56	1	1	3.0
95	95	2256788	2	33	2	1	1.0
96	96	2295324	1	52	3	1	.8
97	97	2296008	1	35	1	1	6.0
98	98	2296367	2	56	2	1	.
99	99	2296655	1	58	3	1	10.0
100	100	2297307	2	69	3	1	7.0
101	101	2297998	2	84	1	1	20.0
102	102	1196362	1	61	1	1	10.0
103	103	1214648	1	65	3	1	10.0
104	104	1232653	2	90	2	1	22.0
105	105	1325524	2	67	3	1	16.0
106	106	1397627	1	63	3	1	11.0
107	107	2257211	1	44	3	1	10.0
108	108	2268268	2	62	2	1	3.0
109	109	2280201	1	67	3	1	10.0
110	110	2298351	1	63	3	1	20.0
111	111	1750345	1	59	3	1	20.0
112	112	1750410	1	57	0	0	.0
113	113	2255591	2	63	1	1	1.0
114	114	2259609	2	70	3	1	50.0
115	115	2264627	2	52	2	1	5.0
116	116	2302015	1	43	3	1	.
117	117	2302649	2	62	3	1	3.0

	CHD	PA	PN	UA	HCY	DNstage	DNstageadjust
79	0	0	1	1	0	3	3
80	0	0	0	0	0	4	4
81	0	1	0	0	0	3	3
82	1	0	1	0	0	4	5
83	0	0	0	1	0	4	4
84	1	1	1	0	0	4	4
85	1	0	0	0	0	5	5
86	0	1	0	0	0	1	4
87	0	1	0	0	0	3	4
88	0	1	0	0	0	2	3
89	0	1	1	0	0	0	4
90	1	0	1	1	0	3	3
91	0	0	1	0	0	5	5
92	0	1	0	1	0	2	4
93	0	0	0	0	1	5	5
94	0	0	0	0	0	0	.
95	0	0	0	1	0	4	4
96	0	0	0	0	0	5	4
97	0	0	0	1	0	2	3
98	0	1	1	0	0	3	1
99	0	0	.	0	1	5	5
100	1	0	0	1	0	3	4
101	0	0	1	0	0	2	2
102	1	0	1	0	0	3	1
103	1	0	1	1	0	3	2
104	0	0	1	0	0	4	3
105	0	0	0	0	0	1	4
106	0	0	1	0	1	4	4
107	1	0	1	0	0	1	3
108	0	1	1	0	0	1	4
109	1	0	1	0	0	4	4
110	0	0	0	0	0	2	3
111	1	0	0	0	0	4	4
112	0	1	0	0	1	1	2
113	0	0	1	0	1	0	4
114	1	1	1	0	0	1	4
115	0	1	1	0	0	4	3
116	0	0	0	0	1	4	4
117	0	1	1	0	0	3	3

	DNstage2	DNstage3	KDeGFR	UPstage	UPstage2	HL
79	1	2	1	2	1	1
80	2	3	2	3	2	1
81	1	2	3	.	.	0
82	2	3	5	3	2	0
83	2	3	3	3	2	1
84	2	3	3	3	2	1
85	2	3	5	.	.	0
86	2	3	1	3	2	1
87	2	3	3	3	2	1
88	1	2	1	2	1	1
89	2	3	4	.	.	0
90	1	2	3	2	1	1
91	2	3	5	.	.	0
92	2	3	2	3	2	1
93	2	3	5	.	.	0
94	.	.	.	.	.	0
95	2	3	4	3	2	1
96	2	3	4	3	2	0
97	1	2	2	2	1	1
98	1	1	1	.	.	1
99	2	3	5	3	2	1
100	2	3	3	3	2	1
101	1	1	2	.	.	0
102	1	1	1	.	.	0
103	1	1	2	.	.	1
104	1	2	3	.	.	1
105	2	3	3	3	2	1
106	2	3	4	3	2	1
107	1	2	1	2	1	1
108	2	3	1	3	2	1
109	2	3	3	3	2	1
110	1	2	1	2	1	0
111	2	3	3	3	2	0
112	1	1	2	1	1	1
113	2	3	2	3	2	1
114	2	3	1	3	2	1
115	1	2	2	2	1	0
116	2	3	3	3	2	1
117	1	2	1	2	1	0

	HLyears	HLmedicine	DMtype	DMyears
79	10.00	1	2	20.0
80	.00	0	2	5.0
81	.00	0	2	20.0
82	.00	0	2	12.0
83	.00	0	2	5.0
84	.00	0	2	17.0
85	.00	0	2	10.0
86	.00	0	2	20.0
87	10.00	4	1	22.0
88	5.00	1	2	10.0
89	.00	0	2	22.0
90	5.00	0	2	19.0
91	.00	0	2	20.0
92	.00	0	2	15.0
93	.00	0	2	20.0
94	.00	0	2	10.0
95	.00	0	2	18.0
96	.00	0	2	5.0
97	.00	0	2	3.0
98	.00	0	2	5.0
99	.00	0	2	16.0
100	.00	0	2	28.0
101	.00	0	2	11.0
102	.00	0	2	28.0
103	.00	0	2	10.0
104	14.00	1	2	22.0
105	.00	0	2	25.0
106	27.00	1	2	.
107	.00	1	2	10.0
108	.00	0	2	16.0
109	.00	0	2	18.0
110	.00	0	2	17.0
111	.00	0	2	20.0
112	.00	0	2	15.0
113	.00	0	2	14.0
114	20.00	0	2	28.0
115	.00	0	2	11.0
116	.00	0	2	.5
117	.00	0	2	17.0

	HbA1c	Height	WeightKg	BMI
79	10.90	170	72.5000	24.9000
80	.	160	49.5500	19.3600
81	.	177	86.0000	27.4500
82	9.90	158	60.0000	24.0300
83	9.10	170	80.0000	27.2000
84	8.00	160	84.5000	33.0000
85	5.30	167	70.0000	25.1000
86	12.60	160	50.0000	19.5300
87	8.20	160	53.0000	20.7000
88	.	170	73.1000	25.3000
89	6.30	175	67.0000	21.8800
90	7.40	160	71.0000	27.7000
91	.	150	57.0000	25.3300
92	10.30	156	60.5000	24.1900
93	5.70	174	98.0000	33.9000
94	9.20	174	76.0000	31.7100
95	9.90	162	80.5000	30.4400
96	4.90	180	87.0000	26.8500
97	.	181	107.0000	32.6200
98	7.90	160	51.5000	21.1000
99	.	170	78.0000	26.9800
100	13.20	158	52.2500	21.3300
101	12.70	160	63.5000	24.8000
102	9.40	180	81.0000	25.0000
103	5.80	167	76.8000	27.5300
104	10.90	153	49.0000	20.9000
105	.	161	76.0000	29.3200
106	16.90	176	86.1000	27.7900
107	7.20	178	115.0000	36.3000
108	7.30	162	65.0000	24.8000
109	6.80	162	65.0000	24.7700
110	7.00	165	80.0000	29.4000
111	.	170	84.0000	29.0700
112	.	169	75.0000	26.2600
113	6.90	154	71.2000	30.0200
114	8.90	160	60.5000	23.7000
115	7.70	158	58.0000	23.2000
116	6.80	168	71.0000	25.2000
117	.	154	56.4500	23.8000

	AC	SBP	DBP	SCRumolL
79	91.0	120	80	68.80
80	83.0	179	93	52.90
81	102.0	155	65	123.50
82	.	153	67	315.20
83	.	147	92	127.50
84	122.0	134	80	10.60
85	.	180	79	971.20
86	.	135	89	52.10
87	.	141	64	104.40
88	92.0	148	91	58.00
89	88.0	123	80	277.30
90	.	130	70	107.20
91	.	126	84	940.40
92	88.0	152	81	84.20
93	124.0	165	93	595.10
94	.	174	88	.
95	103.0	95	68	211.00
96	.	173	106	304.70
97	110.0	139	89	108.40
98	82.0	150	80	56.50
99	.	156	81	449.40
100	80.0	181	91	106.50
101	88.0	154	84	72.30
102	103.0	134	84	61.10
103	.	114	56	79.40
104	94.0	161	72	99.30
105	.	132	68	128.60
106	101.0	153	88	203.20
107	123.0	159	91	72.00
108	.	130	80	49.10
109	.	136	80	145.30
110	102.0	174	87	68.60
111	.	180	99	161.30
112	.	10	82	111.40
113	89.0	160	73	87.20
114	92.0	148	69	52.10
115	84.3	120	80	71.70
116	90.0	120	87	163.40
117	90.0	173	89	46.40

	eGFRmlmin1.73m2	CKDstage	DKDstage	UPstageadj	UAadj
79	94.94	1	3	2	422.00
80	89.97	2	4	3	185.00
81	53.07	3	3	.	265.00
82	13.68	5	5	3	443.00
83	41.45	3	4	3	520.00
84	44.29	3	4	3	389.00
85	5.36	5	5	.	538.00
86	95.65	1	4	3	184.00
87	48.51	3	4	3	395.00
88	121.40	1	3	2	332.00
89	19.41	4	4	.	528.00
90	45.04	3	3	2	381.00
91	3.47	5	5	.	511.00
92	72.49	2	4	3	447.00
93	8.87	5	5	.	436.00
94	.	.	.	.	.
95	25.96	4	4	3	328.00
96	19.38	4	4	3	343.00
97	79.26	2	3	2	365.00
98	99.81	1	1	.	3.90
99	9.55	5	5	3	356.00
100	46.04	3	4	3	422.00
101	82.70	2	2	.	328.00
102	102.53	1	1	.	15.02
103	89.51	2	2	.	289.00
104	43.24	3	3	.	434.00
105	37.18	3	4	3	514.00
106	29.27	4	4	3	313.00
107	108.00	1	3	2	302.00
108	100.31	1	4	3	268.00
109	42.99	3	4	3	374.00
110	96.40	1	3	2	439.00
111	39.80	3	4	3	317.00
112	63.15	2	2	1	288.00
113	61.16	2	4	3	.
114	93.00	1	4	3	304.00
115	83.71	2	3	2	255.00
116	43.85	3	4	3	546.00
117	102.20	1	3	2	417.00

	ACRmgmmol	ACRmgg	UP24hg	CHOLmmolL	TGmmolL
79	12.36	109.26	.	4.63	1.87
80	34.55	305.42	.71	4.45	2.06
81	.	.	8.66	2.79	1.20
82	182.45	1612.86	2.42	4.89	3.71
83	374.65	3311.91	5.05	4.30	2.81
84	800.86	7079.60	.	2.98	2.48
85	.	.	10.55	5.28	2.84
86	103.77	917.33	.	3.56	2.34
87	97.20	859.25	.88	7.84	2.13
88	3.58	31.65	.04	2.98	2.42
89	.	.	.81	.	1.09
90	17.83	157.62	.12	3.18	2.58
91	.	.	.	3.14	1.25
92	421.00	3721.64	3.40	8.29	2.75
93	.	.	15.19	5.03	1.42
94	.	.	.	5.67	9.35
95	434.29	3839.12	9.50	8.58	7.75
96	243.03	2148.39	1.63	4.48	.60
97	4.08	36.07	.	4.41	1.88
98	.	.	.09	4.01	1.65
99	989.12	8743.82	9.56	9.31	4.41
100	73.24	647.44	.	4.83	2.15
101	.	.	.	4.70	1.18
102	.	.	.	2.94	1.29
103	.	.	.	2.45	2.62
104	.	.	.	4.63	1.84
105	177.12	1565.74	4.39	4.53	2.45
106	610.09	5393.20	3.25	7.68	7.74
107	16.02	141.62	.38	3.59	1.64
108	43.28	382.60	.52	3.11	.61
109	48.95	432.72	.18	3.26	1.25
110	4.34	38.37	.12	3.56	1.40
111	266.75	2358.07	2.64	4.12	1.12
112	.94	8.31	.	4.36	1.45
113	63.90	564.88	1.22	5.79	.
114	56.53	499.73	.	4.12	1.11
115	31.71	280.32	.	5.32	1.22
116	97.15	858.81	2.73	3.59	1.60
117	17.68	156.29	.	4.19	1.65

	HDLCmmolL	LDLCmmolL	HCYumolL	GLUmmolL
79	1.02	2.99	.	11.22
80	.50	2.92	13.72	.
81	.96	1.62	.	6.19
82	.90	2.85	23.40	.
83	1.33	2.43	18.12	.
84	.73	1.69	12.41	7.18
85	1.09	3.40	26.84	.
86	1.38	2.71	11.17	15.36
87	1.50	4.82	13.33	.
88	.65	1.76	14.18	8.01
89	1.17	4.07	24.07	.
90	.77	1.82	17.14	8.05
91	1.03	1.74	54.14	.
92	1.24	4.48	.	3.45
93	.85	3.37	18.93	.
94	1.37	3.11	.	.
95	1.17	4.38	15.00	12.33
96	1.09	2.72	31.21	.
97	.89	2.86	21.80	9.70
98	.92	2.56	10.91	8.69
99	1.42	6.14	.	.
100	1.30	3.05	16.81	7.47
101	1.35	2.71	.	5.86
102	1.11	1.55	11.09	7.27
103	.73	1.31	20.25	6.07
104	1.13	2.89	15.49	8.39
105	1.15	2.89	14.83	.
106	.99	4.54	23.64	8.95
107	.89	2.28	11.63	11.99
108	.94	1.82	9.67	.
109	.86	2.10	43.36	8.12
110	1.13	2.04	.	5.99
111	1.09	2.41	34.18	7.84
112	1.48	2.64	24.63	7.23
113	1.09	3.95	11.66	3.16
114	1.19	2.46	13.03	11.31
115	1.23	3.40	11.94	8.56
116	.95	2.15	21.97	.
117	.98	2.66	9.82	6.89

	INSulUml	CPngml	@0.5hGLU	@0.5hINS
79	17.67	1.37	12.82	13.81
80	3.10	.79	.	.
81	11.88	2.49	12.02	20.24
82	.	.	.	.
83	.	.	.	.
84	14.67	3.40	9.09	16.37
85	.	.	.	.
86	6.52	1.25	21.55	14.38
87	.	.	.	.
88	28.08	1.88	8.01	11.61
89	.	.	.	.
90	23.10	7.90	.	.
91	.	.	.	.
92	6.13	.30	6.86	16.05
93	.	.	.	.
94	.	.	.	.
95	13.24	5.20	14.05	26.46
96	.	.	.	.
97	5.58	1.62	10.60	4.03
98	2.83	9.09	7.42	2.30
99	.	.	.	.
100	3.59	.97	.	.
101	3.56	6.21	13.50	4.33
102	19.53	.90	11.71	20.53
103	9.01	1.98	8.62	22.35
104	9.98	1.16	10.17	10.18
105	.	.	.	.
106	24.13	7.67	.	.
107	25.00	4.03	13.59	45.74
108	.	.	.	.
109	.	.	.	.
110	15.42	2.38	11.95	81.86
111	19.05	5.41	8.69	11.32
112	16.56	3.67	13.09	57.86
113	24.01	.37	8.09	33.10
114	9.04	1.30	12.90	2.25
115	17.76	1.65	9.85	14.09
116	.	.	.	.
117	34.22	1.16	12.28	27.06

	@0.5hCP	@1hGLU	@1hINS	@1hCP
79	1.33	16.82	21.45	1.99
80	.	.	.	.
81	3.14	14.70	27.59	4.80
82	.	.	.	.
83	.	.	.	.
84	3.27	13.71	20.83	3.62
85	.	.	.	.
86	2.28	23.80	11.88	2.22
87	.	.	.	.
88	1.60	13.25	10.06	1.82
89	.	.	.	.
90	.	11.14	39.90	9.32
91	.	.	.	.
92	1.44	10.14	22.35	2.95
93	.	.	.	.
94	.	.	.	.
95	5.81	19.92	41.56	7.35
96	.	.	.	.
97	1.51	11.81	4.79	1.59
98	6.94	11.81	2.58	11.50
99	.	.	.	.
100	.	.	.	.
101	?	?	?	?
102	?	?	?	?
103	?	?	?	?
104	?	?	?	?
105	?	?	?	?
106	?	?	?	?
107	?	?	?	?
108	?	?	?	?
109	?	?	?	?
110	?	?	?	?
111	?	?	?	?
112	?	?	?	?
113	?	?	?	?
114	?	?	?	?
115	?	?	?	?
116	?	?	?	?
117	?	?	?	?

	@2hGLU	@2hINS	@2hCP	DRdosmesticR
79	21.57	14.85	1.93	0
80	16.35	.	.	0
81	18.00	38.03	6.31	3
82	.	.	.	2
83	.	.	.	3
84	19.48	34.85	5.44	4
85	.	.	.	0
86	29.70	14.96	2.52	6
87	.	.	.	3
88	17.92	13.99	2.94	0
89	.	.	.	5
90	13.56	43.75	9.83	3
91	.	.	.	6
92	10.81	21.86	3.58	2
93	.	.	.	2
94	.	.	.	4
95	23.92	.	12.16	0
96	.	.	.	3
97	14.84	7.07	1.96	.
98	17.39	3.93	10.34	0
99	.	.	.	3
100	17.45	8.84	1.79	.
101	19.18	4.06	.70	0
102	19.05	33.26	2.91	5
103	15.10	76.94	7.54	0
104	20.96	27.08	2.53	.
105	.	.	.	4
106	.	.	.	4
107	74.21	51.71	6.34	0
108	.	.	.	4
109	.	.	.	5
110	14.30	55.88	8.63	0
111	17.24	22.02	5.34	4
112	15.44	99.07	13.23	0
113	16.15	32.83	4.06	3
114	24.88	26.13	2.23	.
115	12.36	25.45	4.26	3
116	.	.	.	3
117	20.73	30.83	2.56	3

	DRinternatioinalR	DRdandiR	DRdosmesticL	DRinternatioinalL
79	0	0	0	0
80	0	0	0	0
81	2	3	5	4
82	2	2	5	4
83	2	3	2	2
84	4	4	4	4
85	0	0	2	2
86	4	6	6	4
87	2	3	2	2
88	0	0	0	0
89	4	5	4	3
90	2	3	0	0
91	4	6	4	4
92	2	2	2	2
93	1	2	6	4
94	4	4	3	3
95	0	0	2	1
96	2	3	6	4
97	.	.	.	.
98	0	0	2	1
99	2	3	3	2
100	.	.	.	.
101	0	0	3	2
102	4	4	4	4
103	0	0	0	0
104	0	.	0	0
105	4	4	.	.
106	4	4	3	2
107	0	0	0	0
108	3	4	4	3
109	4	5	6	4
110	0	0	0	0
111	3	4	0	0
112	0	0	0	0
113	2	3	0	0
114	.	.	.	.
115	3	3	2	2
116	2	3	0	0
117	2	3	0	0

	DRdandiL	eyechosen	eyechosenD	DRyesorno	DRchosensta
79	0	1	0	0	0
80	0	1	0	0	0
81	4	2	4	1	2
82	4	2	4	1	2
83	2	1	3	1	1
84	4	1	4	1	2
85	2	2	2	1	1
86	6	1	6	1	2
87	2	1	3	1	1
88	0	1	0	0	0
89	4	1	5	1	2
90	0	1	3	1	1
91	4	1	6	1	2
92	2	1	2	1	1
93	6	1	6	1	2
94	3	1	4	1	2
95	2	2	2	1	1
96	6	2	6	1	2
97	.	.	.	.	.
98	2	2	2	1	1
99	3	1	3	1	1
100	.	.	.	.	.
101	3	2	3	1	1
102	4	1	4	1	2
103	0	1	0	0	0
104	0	2	0	0	0
105	.	1	4	1	2
106	3	1	4	1	2
107	0	1	0	0	0
108	4	1	4	1	2
109	6	2	6	1	2
110	0	1	0	0	0
111	0	1	4	1	2
112	0	1	0	0	0
113	0	1	3	1	1
114	.	.	.	.	.
115	2	1	3	1	1
116	0	1	3	1	1
117	0	1	3	1	1

	eyechosened	VA	IOP	cataract
79	1	.80	10.0000	0
80	1	.60	13.0000	0
81	2	.60	14.0000	1
82	2	.	.	0
83	1	.50	12.0000	0
84	1	.60	13.0000	2
85	2	.50	18.0000	1
86	1	.06	22.0000	2
87	1	.40	10.0000	1
88	1	1.00	25.0000	1
89	1	.	9.0000	1
90	1	.25	13.0000	1
91	1	.	12.0000	1
92	1	.40	15.0000	1
93	1	.60	.	3
94	1	.40	12.0000	3
95	2	.60	20.0000	1
96	2	.05	7.0000	0
97	1	1.00	15.0000	1
98	2	.60	14.0000	0
99	1	.25	12.0000	1
100	1	.08	19.0000	1
101	2	.60	15.0000	3
102	1	.80	17.0000	3
103	1	.80	12.0000	0
104	2	.50	13.0000	3
105	1	.80	16.0000	3
106	1	.20	15.0000	0
107	1	1.20	12.0000	0
108	1	1.20	.	0
109	2	.02	11.0000	0
110	1	.25	19.0000	0
111	1	.80	12.0000	0
112	1	.80	16.0000	0
113	1	.15	18.0000	0
114	1	.30	16.0000	.
115	1	.80	13.0000	0
116	1	.80	16.0000	0
117	1	.50	24.0000	0

	MA	Hem	HE	CWspot
79	0	0	0	0
80	0	0	0	0
81	0	1	0	0
82	1	1	1	0
83	1	1	1	1
84	0	1	0	0
85	0	1	1	0
86	.	.	.	.
87	1	1	1	0
88	0	0	0	0
89	0	1	1	0
90	1	1	0	1
91	0	0	0	0
92	0	0	1	0
93	1	0	1	0
94	0	1	1	1
95	0	0	1	0
96	0	0	0	0
97	.	.	.	.
98	0	1	1	0
99	0	1	0	1
100	.	.	.	.
101	0	1	0	1
102	0	0	0	0
103	0	0	0	0
104	0	0	0	0
105	0	0	0	0
106	0	0	0	0
107	0	0	0	0
108	0	0	0	0
109	0	0	0	0
110	0	0	0	0
111	0	0	0	0
112	0	0	0	0
113	0	0	0	1
114	.	.	.	.
115	0	1	1	1
116	0	0	0	1
117	0	0	0	1

	VH	TRD	ME	MEandHE
79	0	0	0	0
80	0	0	0	0
81	1	0	0	0
82	0	0	1	1
83	0	0	0	0
84	0	0	0	0
85	0	0	0	0
86	.	.	.	.
87	0	0	1	1
88	0	0	0	0
89	0	0	0	0
90	0	0	0	0
91	0	1	0	0
92	0	0	1	0
93	0	0	0	0
94	0	0	1	0
95	0	0	0	0
96	0	1	0	0
97	.	.	.	.
98	0	0	0	0
99	0	0	0	0
100	.	.	.	.
101	0	0	0	0
102	1	0	0	0
103	0	0	0	0
104	0	0	0	0
105	0	0	0	0
106	0	0	0	0
107	0	0	0	0
108	0	0	0	0
109	0	1	0	0
110	0	0	0	0
111	0	0	0	0
112	0	0	0	0
113	0	0	0	0
114	.	.	.	.
115	0	0	1	1
116	0	0	0	0
117	0	0	0	0

	DRstaged	DRstagei	DRstagedandi	eyedisothers	eyetreothers
79	0	0	0	0	0
80	0	0	0	0	0
81	5	4	4	0	PPV
82	5	4	4	0	PRP
83	3	2	3	0	0
84	4	4	4	0	PRP
85	2	2	2	0	0
86	6	4	6	0	PPV
87	3	2	3	0	injection
88	0	0	0	0	0
89	5	4	5	0	PPV
90	3	2	3	0	0
91	6	4	6	0	0
92	2	2	2	0	0
93	2	1	2	0	0
94	4	4	4	0	PRP
95	2	1	2	0	0
96	6	4	6	0	0
97	.	.	.		
98	2	1	2	0	0
99	3	2	3	0	0
100	.	.	.		
101	3	2	3	0	0
102	5	4	4	0	PPV
103	0	0	0	0	0
104	0	0	0	drusen	0
105	4	4	4	0	PRP
106	4	4	4	0	PRP
107	0	0	0	0	0
108	4	3	4	0	PRP
109	6	4	6	0	0
110	0	0	0	0	0
111	4	3	4	0	PRP
112	0	0	0	0	0
113	3	2	3	0	0
114	.	.	.		
115	3	3	3	0	0
116	3	2	3	0	0
117	3	2	3	staphyloma	0

	imagequality	HRF	HRFstage	HRFyesorno	HRFlocation
79	1	0	0	0	0
80	1	0	0	0	0
81	1	0	0	0	0
82	1	3	2	1	1
83	1	2	2	1	1
84	1	0	0	0	0
85	1	1	1	1	1
86	0	.	.	.	.
87	1	2	2	1	3
88	1	0	0	0	0
89	1	3	2	1	1
90	1	0	0	0	0
91	0	.	.	.	.
92	1	2	2	1	1
93	0	.	.	.	.
94	1	2	2	1	1
95	1	1	1	1	1
96	0	.	.	.	.
97	.	.	.	.	.
98	1	2	2	1	1
99	1	0	0	0	0
100	.	.	.	.	.
101	1	0	0	0	0
102	1	0	0	0	0
103	1	0	0	0	0
104	1	0	0	0	0
105	1	0	0	0	0
106	1	0	0	0	0
107	1	0	0	0	0
108	1	0	0	0	0
109	0	.	.	.	.
110	1	0	0	0	0
111	1	0	0	0	0
112	1	0	0	0	0
113	1	0	0	0	0
114	.	.	.	.	.
115	1	1	1	1	1
116	1	0	0	0	0
117	0	.	.	.	.

	eyeschosen1R2L	VA2	IOP2	cataract2
79	2	.60	10.0000	0
80	2	.60	13.0000	0
81	2	.60	14.0000	1
82	2	.	.	0
83	2	.40	10.0000	0
84	2	.40	13.0000	2
85	2	.50	18.0000	1
86	2	.40	15.0000	2
87	2	.60	13.0000	1
88	2	1.00	23.0000	1
89	2	.50	8.0000	1
90	2	.25	14.0000	1
91	2	.12	12.0000	1
92	2	.50	18.0000	1
93	2	.	.	3
94	2	.40	12.0000	3
95	2	.60	20.0000	1
96	2	.05	7.0000	0
97	2	.80	15.0000	2
98	2	.60	14.0000	0
99	2	.20	13.0000	1
100	2	.15	17.0000	1
101	2	.60	15.0000	3
102	2	.30	18.0000	1
103	2	.80	15.0000	0
104	2	.50	13.0000	3
105	2	.25	18.0000	1
106	2	.60	15.0000	0
107	2	1.00	12.0000	0
108	2	.80	.	0
109	2	.02	11.0000	0
110	2	.25	20.0000	0
111	2	.40	14.0000	0
112	2	1.00	17.0000	0
113	2	.40	17.0000	0
114	2	.40	14.0000	.
115	2	.60	11.0000	0
116	2	.60	17.0000	0
117	2	.40	27.0000	0

	MA2	Hem2	HE2	CWspot2	VH2
79	0	0	0	0	0
80	0	0	0	0	0
81	0	1	0	0	1
82	1	1	1	0	0
83	1	1	1	0	0
84	0	1	0	0	0
85	0	1	1	0	0
86	.	.	.	.	.
87	1	1	1	0	0
88	0	0	0	0	0
89	0	1	0	0	0
90	0	0	0	0	0
91	0	0	0	0	0
92	0	0	1	0	0
93	0	0	0	0	0
94	0	1	0	0	0
95	0	0	1	0	0
96	0	0	0	0	0
97	.	.	.	.	.
98	0	1	1	0	0
99	0	1	1	1	0
100	.	.	.	.	.
101	0	1	0	1	0
102	0	0	0	0	0
103	0	0	0	0	0
104	0	0	0	0	0
105	.	.	.	.	.
106	0	0	0	1	0
107	0	0	0	0	0
108	0	0	0	0	0
109	0	0	0	0	0
110	0	0	0	0	0
111	0	0	0	0	0
112	0	0	0	0	0
113	0	0	0	0	0
114	.	.	.	.	.
115	0	0	1	0	0
116	0	0	0	0	0
117	0	0	0	0	0

	TRD2	ME2	MEandHE2	DRstaged2
79	0	0	0	0
80	0	0	0	0
81	0	0	0	5
82	0	1	1	5
83	0	0	0	2
84	0	1	0	4
85	0	0	0	2
86	.	.	.	6
87	0	0	0	2
88	0	0	0	0
89	0	1	1	4
90	0	0	0	0
91	0	0	0	4
92	0	1	1	2
93	1	0	0	6
94	0	1	0	3
95	0	0	0	2
96	1	0	0	6
97	.	.	.	.
98	0	0	0	2
99	0	0	0	3
100	.	.	.	.
101	0	0	?	?
102	0	0	?	?
103	0	0	?	?
104	0	0	?	?
105	.	.	?	?
106	0	1	?	?
107	0	0	?	0
108	0	0	0	4
109	1	0	0	6
110	0	0	0	0
111	0	0	0	0
112	0	0	0	0
113	0	0	0	0
114	.	.	.	.
115	0	1	1	2
116	0	0	0	0
117	0	0	0	0

...	DRstagei2	DRstagedandi2	eyedisothers2	eyetreothers2	imagequality2
79	0	0	0	0	1
80	0	0	0	0	1
81	4	4	0	PPV	1
82	4	4	0	PRP	1
83	2	2	2	0	1
84	4	4	0	PRP	1
85	2	2	0	0	1
86	4	6	0	PPV	.
87	2	2	0	0	1
88	0	0	0	0	1
89	3	4	0	PRP	1
90	0	0	MH	0	0
91	4	4	0	PRP	0
92	2	2	0	0	1
93	4	6	0	PPV	0
94	3	3	0	injection	1
95	1	2	0	0	1
96	4	6	0	0	0
97	.	.			.
98	1	2	0	0	1
99	2	3	0	0	1
100	.	.			.
101	2	3	0	0	1
102	4	4	ERM	PRP	1
103	0	0	0	0	1
104	0	0	drusen	0	1
105	.	.			0
106	2	3	0	0	1
107	0	0	0	0	1
108	3	4	0	PRP	1
109	4	6	0	0	0
110	0	0	0	0	1
111	0	0	0	0	1
112	0	0	astro	0	1
113	0	0	0	0	1
114	.	.			.
115	2	2	0	0	1
116	0	0	0	0	1
117	0	0	staphyloma	0	0

	HRFstage2	HRFlocation2	filter_$	ln_acr	ln_egfr
79	0	0	0	4.69	4.55
80	0	0	0	5.72	4.50
81	0	0	0	.	3.97
82	3	1	1	7.39	2.62
83	3	1	1	8.11	3.72
84	2	1	0	8.86	3.79
85	1	1	1	.	1.68
86	.	.	.	6.82	4.56
87	2	1	1	6.76	3.88
88	0	0	0	3.45	4.80
89	2	1	1	.	2.97
90	.	.	0	5.06	3.81
91	.	.	0	.	1.24
92	3	3	1	8.22	4.28
93	.	.	1	.	2.18
94	2	1	1	.	.
95	1	1	1	8.25	3.26
96	.	.	0	7.67	2.96
97	.	.	.	3.59	4.37
98	2	1	1	.	4.60
99	1	1	0	9.08	2.26
100	.	.	.	6.47	3.83
101	0	0	0	.	4.42
102	0	0	0	.	4.63
103	0	0	0	.	4.49
104	0	0	0	.	3.77
105	.	.	0	7.36	3.62
106	0	0	0	8.59	3.38
107	0	0	0	4.95	4.68
108	2	1	0	5.95	4.61
109	.	.	0	6.07	3.76
110	0	0	0	3.65	4.57
111	0	0	0	7.77	3.68
112	0	0	0	2.12	4.15
113	0	0	0	6.34	4.11
114	.	.	.	6.21	4.53
115	2	1	1	5.64	4.43
116	0	0	0	6.76	3.78
117	.	.	0	5.05	4.63

	egfrgroup4	egfrgroup3	malb2	LogMAR
79	1.00	1.00	1.00	.22
80	2.00	2.00	2.00	.51
81	3.00	3.00	.	.51
82	4.00	3.00	2.00	.
83	3.00	3.00	2.00	.69
84	3.00	3.00	2.00	.51
85	4.00	3.00	.	.69
86	1.00	1.00	2.00	2.81
87	3.00	3.00	2.00	.92
88	1.00	1.00	1.00	.00
89	4.00	3.00	.	.
90	3.00	3.00	1.00	1.39
91	4.00	3.00	.	.
92	2.00	2.00	2.00	.92
93	4.00	3.00	.	.51
94	.	.	.	.92
95	4.00	3.00	2.00	.51
96	4.00	3.00	2.00	3.00
97	2.00	2.00	1.00	.00
98	1.00	1.00	.	.51
99	4.00	3.00	2.00	1.39
100	3.00	3.00	2.00	2.53
101	2.00	2.00	.	.51
102	1.00	1.00	.	.22
103	2.00	2.00	.	.22
104	3.00	3.00	.	.69
105	3.00	3.00	2.00	.22
106	4.00	3.00	2.00	1.61
107	1.00	1.00	1.00	-.18
108	1.00	1.00	2.00	-.18
109	3.00	3.00	2.00	3.91
110	1.00	1.00	1.00	1.39
111	3.00	3.00	2.00	.22
112	2.00	2.00	1.00	.22
113	2.00	2.00	2.00	1.90
114	1.00	1.00	2.00	1.20
115	2.00	2.00	1.00	.22
116	3.00	3.00	2.00	.22
117	1.00	1.00	1.00	.69

	No	ID	Sex	Age	HBPstage	HBP	HBPyears
118	118	2302492	1	65	0	0	.0
119	119	2303503	1	75	3	1	35.0
120	120	2303520	1	54	3	1	1.0
121	121	2304206	1	61	1	1	10.0
122	122	2304561	2	52	3	1	.3
123	123	2304645	1	67	2	1	15.0
124	124	2305274	1	64	3	1	20.0
125	125	2298774	2	42	3	1	7.0
126	126	2299825	2	55	1	1	3.0
127	127	2300019	1	47	3	1	1.0
128	128	2300641	2	71	3	1	15.0
129	129	2300717	1	56	3	1	.5
130	130	2301553	2	71	1	1	10.0
131	131	2302274	1	61	2	1	.
132	132	1068133	1	63	1	1	20.0
133	133	1105685	1	86	1	1	.
134	134	1180573	1	82	1	1	.
135	135	1273462	2	71	0	0	.0
136	136	1305814	1	59	2	1	20.0
137	137	1338062	2	49	0	0	.0
138	138	1376535	1	86	1	1	10.0
139	139	1399764	1	46	1	1	.3
140	140	1405726	1	59	3	1	2.0
141	141	1454828	2	59	3	1	7.0
142	142	1456972	2	55	0	0	.0
143	143	1459120	1	57	3	1	16.0
144	144	1501719	1	42	3	1	1.0
145	145	1750296	1	56	3	1	5.0

	CHD	PA	PN	UA	HCY	DNstage	DNstageadjust
118	0	1	1	0	0	1	3
119	1	0	1	1	0	3	2
120	0	1	0	0	0	4	4
121	0	1	0	1	0	.	3
122	0	0	0	0	0	4	4
123	1	0	0	0	1	1	3
124	1	1	1	1	0	.	1
125	0	0	0	0	0	4	4
126	0	1	1	0	0	4	4
127	0	0	1	1	0	2	4
128	1	1	1	0	0	3	3
129	0	0	0	0	0	4	4
130	1	0	1	0	0	1	4
131	0	1	0	0	0	0	3
132	0	1	1	1	0	.	3
133	1	0	1	1	0	1	3
134	0	0	1	1	1	1	4
135	0	1	1	0	0	1	3
136	1	0	1	1	0	3	3
137	0	1	1	0	0	1	4
138	0	1	1	0	1	3	4
139	0	1	0	0	0	1	1
140	0	1	0	0	0	3	3
141	0	1	1	0	0	1	3
142	1	0	0	1	0	1	4
143	1	1	1	0	0	3	3
144	0	0	1	0	1	3	4
145	0	1	1	0	0	3	3

	DNstage2	DNstage3	KDeGFR	UPstage	UPstage2	HL
118	1	2	1	2	1	1
119	1	1	2	.	.	1
120	2	3	3	3	2	1
121	1	2	1	2	1	1
122	2	3	2	3	2	1
123	1	2	1	2	1	1
124	1	1	1	1	1	1
125	2	3	2	3	2	0
126	2	3	1	3	2	0
127	2	3	2	3	2	1
128	1	2	3	2	1	0
129	2	3	1	3	2	1
130	2	3	2	3	2	1
131	1	2	1	2	1	1
132	1	2	3	1	1	1
133	1	2	3	2	1	1
134	2	3	3	3	2	0
135	1	2	1	2	1	1
136	1	2	1	2	1	1
137	2	3	1	3	2	1
138	2	3	3	3	2	1
139	1	1	1	1	1	1
140	1	2	1	2	1	1
141	1	2	1	2	1	1
142	2	3	1	3	2	1
143	1	2	1	2	1	1
144	2	3	3	3	2	0
145	1	2	2	2	1	1

	HLyears	HLmedicine	DMtype	DMyears
118	.00	0	2	.2
119	.00	1	2	26.0
120	.00	1	2	6.0
121	10.00	1	2	10.0
122	.00	0	2	5.0
123	.00	1	2	17.0
124	30.00	1	2	.1
125	.00	0	2	7.0
126	.00	0	2	11.0
127	.00	0	2	14.0
128	.00	0	2	18.0
129	.00	0	2	8.0
130	5.00	1	2	15.0
131	.00	0	2	14.0
132	.00	0	2	17.0
133	.00	0	2	26.0
134	.00	0	2	42.0
135	9.00	0	2	20.0
136	.00	0	2	2.0
137	.00	0	2	14.0
138	.00	0	2	40.0
139	.30	0	2	7.0
140	3.00	0	2	20.0
141	7.00	1	2	24.0
142	.00	0	2	18.0
143	.00	0	2	11.0
144	.00	0	2	1.0
145	.00	0	2	.2

	HbA1c	Height	WeightKg	BMI
118	6.60	174	51.2000	16.9100
119	8.60	175	78.0000	25.4700
120	6.50	171	94.7000	32.4000
121	7.00	170	67.5000	23.3600
122	10.70	160	64.2000	25.0000
123	8.70	173	83.0000	27.7000
124	5.80	167	76.9000	27.6000
125	7.80	162	73.0000	27.8000
126	6.40	160	53.7000	20.9800
127	9.60	176	86.0000	28.0000
128	8.60	150	61.0000	27.1000
129	9.70	170	75.0000	25.9500
130	.	162	66.5000	.
131	8.10	168	63.2000	22.3900
132	8.80	170	85.0000	29.4000
133	10.00	170	69.0000	23.0000
134	8.70	176	73.3000	23.7600
135	11.70	155	59.9000	24.9000
136	.	170	83.2000	27.4800
137	10.80	173	60.6000	20.2500
138	.	170	61.0000	20.3800
139	6.20	176	77.0000	24.8000
140	.	171	85.0000	29.0600
141	7.70	176	88.0000	28.4000
142	9.60	164	91.1000	33.7000
143	8.20	176	88.0000	28.4000
144	6.70	165	72.0000	26.4500
145	.	172	79.0000	26.7000

renamed_63cd7 12.10 submit.sav

	AC	SBP	DBP	SCRumolL
118	75.0	107	73	74.40
119	.	140	75	95.10
120	110.0	147	90	140.30
121	.	140	88	79.60
122	94.0	136	85	68.40
123	107.0	130	70	81.90
124	98.0	143	74	80.30
125	.	197	102	102.00
126	80.0	155	84	64.50
127	95.0	127	78	107.80
128	.	116	66	88.00
129	.	112	74	81.40
130	90.0	143	69	63.10
131	89.0	162	97	62.40
132	101.0	137	87	114.10
133	83.0	120	61	138.80
134	88.0	170	78	122.00
135	92.0	127	63	52.50
136	95.0	164	85	79.00
137	83.0	169	94	65.20
138	98.0	129	61	114.10
139	99.0	119	80	55.00
140	103.0	143	83	52.00
141	.	144	77	72.70
142	111.0	150	90	54.00
143	102.0	125	76	68.30
144	.	182	99	149.10
145	98.0	158	108	100.60

	eGFRmlmin1.73m2	CKDstage	DKDstage	UPstageadj	UAadj
118	91.94	1	3	2	408.00
119	67.38	2	2	.	497.00
120	48.80	3	4	3	.
121	91.97	1	3	2	452.00
122	73.10	2	4	3	399.00
123	90.11	1	3	2	378.00
124	116.94	1	1	1	399.00
125	61.70	2	4	3	378.00
126	93.15	1	4	3	489.00
127	70.73	2	4	3	452.00
128	57.18	3	3	2	307.00
129	92.93	1	4	3	288.00
130	86.90	2	4	3	356.00
131	101.65	1	3	2	229.00
132	58.81	3	3	1	270.00
133	39.48	3	3	2	155.00
134	47.46	3	4	3	321.00
135	92.12	1	3	2	269.00
136	94.22	1	3	2	229.00
137	95.90	1	4	3	404.00
138	50.04	3	4	3	370.00
139	119.79	1	1	1	290.00
140	111.11	1	3	2	331.00
141	97.49	1	3	2	354.00
142	102.12	1	4	3	528.00
143	101.44	1	3	2	402.00
144	37.58	3	4	3	348.00
145	72.44	2	3	2	364.00

	ACRmgmmol	ACRmgg	UP24hg	CHOLmmolL	TGmmolL
118	3.54	31.29	.	3.74	.59
119	.	.	.	3.58	1.04
120	477.79	4223.66	8.48	4.11	1.61
121	6.06	53.57	.	2.54	1.50
122	77.86	688.28	.49	4.57	2.26
123	4.16	36.77	.	2.80	1.89
124	.17	1.50	.	3.49	2.13
125	105.36	931.38	1.86	5.93	1.16
126	617.40	5457.82	.	4.40	2.27
127	75.15	664.33	.	6.83	1.63
128	7.74	68.42	.19	3.90	1.07
129	220.75	1951.43	.	5.34	4.33
130	219.37	1939.23	1.91	6.80	2.72
131	18.19	160.80	.14	4.38	1.60
132	1.68	14.85	.	3.60	2.15
133	4.02	35.54	.08	3.26	2.42
134	188.92	1670.05	2.97	3.94	.45
135	7.16	63.29	.	4.44	.92
136	28.21	249.38	.15	4.37	2.34
137	314.95	2784.16	4.41	6.07	1.19
138	435.15	3846.73	3.98	4.34	1.42
139	1.92	16.97	.	4.20	1.56
140	17.33	153.20	.27	3.14	.87
141	10.34	91.41	.23	1.74	1.20
142	46.06	407.17	.	5.01	2.76
143	4.88	43.14	.08	3.09	3.51
144	424.25	3750.37	7.26	6.91	8.08
145	33.79	298.70	.	4.11	.88

renamed_63cd7 12.10 submit.sav

	HDLCmmolL	LDLCmmolL	HCYumolL	GLUmmolL
118	1.35	2.18	13.90	5.51
119	.90	2.23	11.65	.
120	.92	2.59	17.77	6.94
121	.57	1.66	10.50	.
122	.89	3.15	12.77	11.30
123	.77	1.73	15.42	7.54
124	.74	2.25	11.19	6.39
125	1.40	3.85	15.43	9.40
126	.74	2.83	10.17	6.54
127	1.26	4.46	17.07	4.16
128	.96	2.57	16.63	.
129	.98	3.33	16.02	7.21
130	1.36	4.25	7.76	5.32
131	.88	2.96	11.04	7.01
132	.75	2.21	13.49	7.09
133	.66	2.12	.	17.87
134	2.31	1.81	14.83	8.82
135	.89	2.91	7.84	7.58
136	1.04	2.78	18.14	9.15
137	1.13	4.14	15.31	5.31
138	.95	2.66	13.95	6.17
139	1.49	2.25	14.17	8.14
140	1.00	1.87	12.59	7.85
141	.52	1.05	11.93	7.18
142	1.12	3.12	11.78	10.86
143	.78	1.85	11.06	12.32
144	1.10	3.67	16.74	11.70
145	.99	2.70	15.91	13.51

	INSuIUml	CPngml	@0.5hGLU	@0.5hINS
118	3.36	1.43	6.34	4.00
119	.	.	.	.
120	7.94	4.15	8.12	13.88
121	.	.	.	.
122	14.16	2.22	12.25	17.03
123	20.24	2.00	12.05	25.20
124	13.79	3.83	10.18	64.64
125	9.08	2.06	9.49	19.09
126	2.24	1.30	12.80	6.90
127	24.22	6.57	4.68	17.81
128	14.01	4.25	.	.
129	8.11	1.24	9.24	7.43
130	6.27	.71	9.69	14.10
131	5.17	2.16	9.40	9.41
132	5.55	2.01	11.89	10.42
133	28.39	1.93	19.11	59.64
134	4.57	.57	13.62	5.57
135	3.18	.38	10.27	7.00
136	10.56	1.33	12.19	13.44
137	9.60	.64	6.36	8.19
138	3.28	1.12	11.64	3.89
139	18.70	2.30	7.86	19.12
140	7.05	1.61	14.24	12.43
141	294.70	2.19	.	.
142	25.18	2.84	14.36	28.87
143	13.01	3.64	13.15	13.28
144	15.83	3.07	12.61	15.63
145	19.60	6.14	9.62	11.00

	@0.5hCP	@1hGLU	@1hINS	@1hCP
118	1.40	11.42	13.31	2.85
119	.	.	.	.
120	4.67	10.39	20.56	5.66
121	.	.	.	.
122	2.65	15.81	21.76	3.12
123	2.65	16.56	28.05	3.14
124	6.99	11.67	75.63	8.72
125	3.32	14.36	32.03	5.12
126	2.25	17.79	.72	2.65
127	.86	5.04	15.70	.80
128	.	.	.	.
129	1.14	14.43	9.54	1.32
130	1.88	13.36	18.30	2.74
131	2.52	16.03	16.73	3.46
132	1.71	17.52	23.51	3.89
133	1.97	24.40	72.13	2.40
134	.39	15.62	8.00	1.41
135	.77	14.52	9.88	1.40
136	1.65	13.76	11.25	1.59
137	.72	8.67	9.99	.99
138	1.08	17.18	6.64	1.41
139	2.19	10.63	28.63	2.92
140	1.96	18.04	24.60	2.93
141	.	9.96	300.40	2.54
142	3.16	15.83	32.37	3.41
143	3.84	14.29	18.63	4.36
144	3.05	12.66	14.82	2.93
145	4.40	14.55	18.44	4.57

	@2hGLU	@2hINS	@2hCP	DRdosmesticR
118	11.63	17.56	5.80	.
119	.	.	.	.
120	12.93	26.95	7.34	0
121	.	.	.	0
122	19.98	27.79	3.88	3
123	21.01	42.07	4.65	0
124	12.64	122.70	13.51	0
125	13.35	39.04	7.30	4
126	22.23	12.79	3.74	3
127	7.00	16.60	1.21	4
128	.	.	.	0
129	17.23	15.00	2.42	0
130	15.27	21.22	3.86	0
131	18.42	19.46	6.03	0
132	17.52	35.32	5.97	0
133	31.00	87.62	2.55	0
134	19.66	9.00	1.45	0
135	19.82	10.20	1.72	5
136	15.40	12.40	1.70	6
137	13.96	13.60	1.75	6
138	23.30	7.88	1.98	0
139	11.25	33.22	4.08	0
140	19.40	37.21	5.45	0
141	10.39	277.20	2.68	6
142	19.25	30.07	3.33	0
143	18.11	31.41	6.20	0
144	1.75	.	3.23	6
145	17.53	22.83	5.24	3

	DRinternatioinalR	DRdandiR	DRdosmesticL	DRinternatioinalL
118	0	.	0	0
119	.	.	.	.
120	0	0	0	0
121	0	0	0	0
122	3	3	2	2
123	0	0	0	0
124	0	0	0	0
125	3	4	5	4
126	2	3	2	2
127	3	4	0	0
128	0	0	.	.
129	0	0	0	0
130	0	0	0	0
131	0	0	0	0
132	0	0	0	0
133	0	0	0	0
134	0	0	0	0
135	4	4	5	4
136	4	6	0	0
137	4	6	5	4
138	0	0	0	0
139	0	0	0	0
140	0	0	0	0
141	4	6	?	?
142	0	0	?	?
143	0	0	?	?
144	4	6	?	?
145	2	3	?	2

	DRdandiL	eyechosen	eyechosenD	DRyesorno	DRchosensta
118	0	2	0	0	0
119	.	.	.	.	.
120	0	1	0	0	0
121	0	1	0	0	0
122	2	1	3	1	1
123	0	1	0	0	0
124	0	1	0	0	0
125	5	1	5	1	2
126	2	1	2	1	1
127	0	1	4	1	2
128	.	1	0	0	0
129	0	1	0	0	0
130	0	1	0	0	0
131	0	1	0	0	0
132	0	1	0	0	0
133	0	1	0	0	0
134	0	1	0	0	0
135	4	1	4	1	2
136	0	1	6	1	2
137	5	1	6	1	2
138	0	1	0	0	0
139	0	1	0	0	0
140	0	1	0	0	0
141	5	1	6	1	2
142	3	2	3	1	1
143	0	1	0	0	0
144	4	1	6	1	2
145	3	1	3	1	1

renamed_63cd7 12.10 submit.sav

	eyechosened	VA	IOP	cataract
118	2	1.00	23.0000	1
119	.	.	.	.
120	1	.80	15.0000	0
121	1	.	.	0
122	1	.60	14.0000	0
123	1	.80	20.0000	0
124	1	.80	12.0000	0
125	1	.50	18.0000	1
126	1	.40	19.0000	0
127	1	.15	14.0000	2
128	1	.60	12.0000	0
129	1	1.00	11.0000	0
130	1	.40	17.0000	0
131	1	.50	17.0000	0
132	1	1.00	12.0000	0
133	1	.60	12.0000	0
134	1	1.00	15.0000	0
135	1	.02	18.0000	0
136	1	.80	14.0000	2
137	1	.10	19.0000	1
138	1	.50	22.0000	0
139	1	1.00	19.0000	0
140	1	1.00	15.0000	0
141	1	.80	12.0000	?
142	2	.60	20.0000	?
143	1	.50	14.0000	?
144	1	.15	13.0000	?
145	1	.80	14.0000	?

	MA	Hem	HE	CWspot
118	0	0	0	0
119	.	.	.	.
120	0	0	0	0
121	0	0	0	0
122	0	1	1	1
123	0	0	0	0
124	0	0	0	0
125	0	0	0	0
126	0	1	1	1
127	0	0	0	0
128	0	0	0	0
129	0	0	0	0
130	0	0	0	0
131	0	0	0	0
132	0	0	0	0
133	0	0	0	0
134	0	0	0	0
135	0	0	1	0
136	0	0	0	0
137	0	0	0	0
138	0	0	0	0
139	0	0	0	0
140	0	0	0	0
141	0	0	0	0
142	0	0	0	1
143	0	0	0	0
144	0	0	0	0
145	0	0	0	1

	VH	TRD	ME	MEandHE
118	0	0	0	0
119	.	.	.	.
120	0	0	0	0
121	0	0	0	0
122	0	0	1	1
123	0	0	0	0
124	0	0	0	0
125	0	0	0	0
126	0	0	1	1
127	0	0	0	0
128	0	0	0	0
129	0	0	0	0
130	0	0	0	0
131	0	0	0	0
132	0	0	0	0
133	0	0	0	0
134	0	0	0	0
135	1	0	0	0
136	0	1	0	0
137	0	1	0	0
138	0	0	0	0
139	0	0	0	0
140	0	0	0	0
141	0	1	0	0
142	0	0	0	0
143	0	0	0	0
144	0	1	0	0
145	0	0	0	0

	DRstaged	DRstagei	DRstagedandi	eyedisothers	eyetreothers
118	0	0	0	0	0
119	.	.	.		
120	0	0	0	0	0
121	0	0	0	0	0
122	3	3	3	0	0
123	0	0	0	0	0
124	0	0	0	0	0
125	4	3	4	0	PRP
126	3	2	3	0	0
127	4	3	4	ERM	PRP
128	0	0	0	0	0
129	0	0	0	0	0
130	0	0	0	0	0
131	0	0	0	0	0
132	0	0	0	0	0
133	0	0	0	0	0
134	0	0	0	0	0
135	5	4	4	0	0
136	6	4	6	0	PPV
137	6	4	6	0	0
138	0	0	0	0	0
139	0	0	0	0	0
140	0	0	0	0	0
141	6	?	?	?	?
142	3	?	?	?	?
143	0	?	?	?	?
144	6	?	?	?	?
145	3	?	?	?	?

	imagequality	HRF	HRFstage	HRFyesorno	HRFlocation
118	1	0	0	0	0
119	0	.	.	.	.
120	1	.	.	.	.
121	1	0	0	0	0
122	1	2	2	1	3
123	0	.	.	.	.
124	1	.	.	.	.
125	1	0	0	0	0
126	1	2	2	1	1
127	0	.	.	.	.
128	1	0	0	0	0
129	1	0	0	0	0
130	1	0	0	0	0
131	1	.	.	.	.
132	1	0	0	0	0
133	1	0	0	0	0
134	1	0	0	0	0
135	0	.	.	.	.
136	0	.	.	.	.
137	0	.	.	.	.
138	1	0	0	0	0
139	0	.	.	.	.
140	1	0	0	0	0
141	1	0	0	0	0
142	1	1	1	1	1
143	1	.	.	.	.
144	0	.	.	.	.
145	1	0	0	0	0

	eyeschosen1R2L	VA2	IOP2	cataract2
118	2	1.00	23.0000	1
119	.	.	.	.
120	2	.40	13.0000	0
121	2	.	.	0
122	2	.40	15.0000	0
123	2	1.20	21.0000	0
124	2	.60	14.0000	0
125	2	.50	17.0000	1
126	2	.25	17.0000	0
127	2	.80	15.0000	1
128	2	.	.	.
129	2	.80	10.0000	0
130	2	.40	15.0000	0
131	2	.60	18.0000	0
132	.	2.00	13.0000	0
133	2	.80	13.0000	0
134	2	.80	13.0000	0
135	2	.02	17.0000	0
136	2	.60	18.0000	2
137	.	.10	20.0000	1
138	2	.60	23.0000	0
139	2	1.00	18.0000	0
140	2	1.00	14.0000	0
141	2	?	?	?
142	2	?	?	?
143	2	?	?	?
144	2	?	?	?
145	2	?	?	?

	MA2	Hem2	HE2	CWspot2	VH2
118	0	0	0	0	0
119	.	.	.	.	.
120	0	0	0	1	0
121	0	0	0	0	0
122	1	1	1	0	0
123	0	0	0	0	0
124	0	0	0	0	0
125	0	0	0	0	0
126	0	1	1	0	0
127	0	0	0	0	0
128	.	.	.	.	.
129	0	0	0	0	0
130	0	0	0	0	0
131	0	0	0	0	0
132	0	0	0	0	0
133	0	0	0	0	0
134	0	0	0	0	0
135	0	0	0	0	1
136	0	0	0	0	0
137	0	0	1	0	1
138	0	0	0	0	0
139	0	0	0	0	0
140	0	0	0	0	0
141	0	0	0	0	1
142	0	0	0	1	0
143	0	0	0	0	0
144	0	0	0	0	1
145	0	1	0	1	0

renamed_63cd7 12.10 submit.sav

	TRD2	ME2	MEandHE2	DRstaged2
118	0	0	0	0
119	.	.	.	.
120	0	0	0	0
121	0	0	0	0
122	0	1	1	2
123	0	0	0	0
124	0	0	0	0
125	0	0	0	5
126	0	1	1	2
127	0	0	0	0
128	.	.	.	.
129	0	0	0	0
130	0	0	0	0
131	0	0	0	0
132	0	0	0	0
133	0	0	0	0
134	0	0	0	0
135	0	0	0	5
136	0	0	0	0
137	0	0	0	5
138	0	0	0	0
139	0	0	0	0
140	0	0	0	0
141	0	1	0	5
142	0	0	0	3
143	0	0	0	0
144	0	1	0	4
145	0	0	0	3

	DRstagei2	DRstagedandi2	eyedisothers2	eyetreothers2	imagequal[illegible]
118	0	0	0	0	1
119	.	.			0
120	0	0	0	0	1
121	0	0	0	0	1
122	2	2	0	0	1
123	0	0	0	0	0
124	0	0	0	0	1
125	4	5	0	PPV	0
126	2	2	PED	0	1
127	0	0	0	0	1
128	.	.			.
129	0	0	0	0	1
130	0	0	0	0	1
131	0	0	0	0	1
132	0	0	0	0	1
133	0	0	0	0	1
134	0	0	0	0	1
135	4	4	0	0	0
136	0	0	0	0	1
137	4	5	0	0	0
138	0	0	0	0	1
139	0	0	0	0	0
140	0	0	0	0	1
141	4	5	0	injection	1
142	3	3	0	PRP	1
143	0	0	0	0	1
144	4	4	0	PRP	0
145	2	3	0	0	1

	HRFstage2	HRFlocation2	filter_$	ln_acr	ln_egfr
118	0	0	0	3.44	4.52
119	.	.	.	.	4.21
120	.	.	0	8.35	3.89
121	0	0	0	3.98	4.52
122	2	3	1	6.53	4.29
123	.	.	0	3.60	4.50
124	.	.	0	.41	4.76
125	.	.	0	6.84	4.12
126	2	1	1	8.60	4.53
127	0	0	0	6.50	4.26
128	.	.	0	4.23	4.05
129	0	0	0	7.58	4.53
130	0	0	0	7.57	4.46
131	.	.	0	5.08	4.62
132	0	0	0	2.70	4.07
133	0	0	0	3.57	3.68
134	0	0	0	7.42	3.86
135	.	.	1	4.15	4.52
136	0	0	0	5.52	4.55
137	.	.	0	7.93	4.56
138	0	0	0	8.25	3.91
139	.	.	0	2.83	4.79
140	0	0	0	5.03	4.71
141	0	0	0	4.52	4.58
142	1	1	0	6.01	4.63
143	.	.	0	3.76	4.62
144	.	.	0	8.23	3.63
145	0	0	0	5.70	4.28

renamed_63cd7 12.10 submit.sav

	egfrgroup4	egfrgroup3	malb2	LogMAR
118	1.00	1.00	1.00	.00
119	2.00	2.00	.	.
120	3.00	3.00	2.00	.22
121	1.00	1.00	1.00	.
122	2.00	2.00	2.00	.51
123	1.00	1.00	1.00	.22
124	1.00	1.00	1.00	.22
125	2.00	2.00	2.00	.69
126	1.00	1.00	2.00	.92
127	2.00	2.00	2.00	1.90
128	3.00	3.00	1.00	.51
129	1.00	1.00	2.00	.00
130	2.00	2.00	2.00	.92
131	1.00	1.00	1.00	.69
132	3.00	3.00	1.00	.00
133	3.00	3.00	1.00	.51
134	3.00	3.00	2.00	.00
135	1.00	1.00	1.00	3.91
136	1.00	1.00	1.00	.22
137	1.00	1.00	2.00	2.30
138	3.00	3.00	2.00	.69
139	1.00	1.00	1.00	.00
140	1.00	1.00	1.00	.00
141	1.00	1.00	1.00	.22
142	1.00	1.00	2.00	.51
143	1.00	1.00	1.00	.69
144	3.00	3.00	2.00	1.90
145	2.00	2.00	1.00	.22
