# Supplementary material for: Retinal microvascular changes in diabetic patients with diabetic nephropathy
Source: BMC Endocr Disord. 2023 May 5;23:101. doi: 10.1186/s12902-022-01250-w (PMC10161482; doi:10.1186/s12902-022-01250-w)
Supplement: Supplementary file 2 — Additional file 2. [file 12902_2022_1250_MOESM2_ESM.docx]

**Additional File 2: Demographic and clinical characteristics in DME and Non-DME groups**

| Variable | DME  N=13 | Non-DME  N=125 | P value |
| --- | --- | --- | --- |
| Sex, N (%) | | | 0.229 |
| Male | 6/13 (46.2) | 79/125 (63.2) |  |
| Female | 7/13 (53.8) | 46/125 (36.8) |  |
| Age, years, mean ± SD | 58.54 ± 8.33 | 60.51 ± 13.04 | 0.595 |
| Visual acuity, LogMAR, mean ± SD | 0.42 ± 0.15 | 0.61 ± 0.28 | 0.003* |
| IOP, mmHg, mean ± SD | 13.08 ± 2.97 | 15.06 ± 3.68 | 0.074 |
| DR severity scale, N (%) |  |  |  |
| None | 0/13 (0) | 62/125 (49.6) | 0.001* |
| Mild NPDR | 1/13 (7.7) | 8/125 (6.4) |  |
| Moderate NPDR | 8/13 (61.5) | 24/125 (19.2) |  |
| SNPDR | 2/13 (15.4) | 5/125 (4.0) |  |
| PDR | 2/13 (15.4) | 26/125 (20.8) |  |
| BMI, kg/m^2^, mean ± SD | 24.33 ± 2.88 | 26.63 ± 4.02 | 0.047* |
| Abdominal circumference, cm, mean ± SD | 88.26 ± 6.19 | 96.72 ± 12.65 | 0.085 |
| Systolic pressure, mmHg, mean ± SD | 151.00 ± 20.16 | 140.98 ± 28.54 | 0.220 |
| Diastolic pressure, mmHg, mean ± SD | 76.08 ± 8.76 | 79.91 ± 11.62 | 0.250 |
| Stage of hypertension, N (%) | | | 0.600 |
| Normal blood pressure | 1/13 (7.7) | 26/124 (21.0) |  |
| Stage 1 | 5/13 (38.5) | 31/124 (25.0) |  |
| Stage 2 | 2/13 (15.4) | 20/124 (16.1) |  |
| Stage 3 | 5/13 (38.5) | 47/124 (37.9) |  |
| Serum lipid profiles |  |  |  |
| Total cholesterol, mmol/L, mean ± SD | 5.00 ± 1.55 | 4.36 ± 1.50 | 0.145 |
| Triglycerides, mmol/L, mean ± SD | 2.63 ± 2.20 | 2.60 ± 3.67 | 0.973 |
| HDL cholesterol, mmol/L, mean ± SD | 1.06 ± 0.24 | 1.06 ± 0.47 | 1.000 |
| LDL cholesterol, mmol/L, mean ± SD | 3.34 ± 0.81 | 2.61 ± 0.94 | 0.020^*^ |
| History of hyperlipidemia, N (%) | | | 0.203 |
| No | 6/31 (46.2) | 36/124 (29.0) |  |
| Yes | 7/31 (53.8) | 88/124 (71.0) |  |
| History of hyperuricemia, N (%) | | | 0.425 |
| No | 11/13 (84.6) | 96/124 (77.4) |  |
| Yes | 2/13 (15.4) | 28/124 (22.6) |  |
| History of hyperhomocystinemia, N (%) | | | 0.499 |
| No | 12/13 (92.3) | 108/124 (87.1) |  |
| Yes | 1/13 (7.7) | 16/124 (12.9) |  |
| Serum uric acid, μmol/L, mean ± SD | 396.33 ± 74.64 | 361.87 ± 108.17 | 0.284 |
| Serum HCY, mmol/L, mean ± SD | 14.86 ± 4.33 | 15.73 ± 7.29 | 0.700 |
| Duration of DM, years, mean ± SD | 12.92 ± 6.03 | 15.09 ± 8.72 | 0.384 |
| HbA1c, %, mean ± SD | 8.40 ± 1.30 | 8.80 ± 2.13 | 0.513 |
| ACR, mg/g, median (interquartile range) | 1236.05  (268.01, 4155.68) | 239.92  (56.13, 790.47) | 0.009^*^ |
| Stage of urine microalbumin, N (%) | | | 0.117 |
| Stage 1&2 | 3/10 (30.0) | 60/109 (55.0) |  |
| Stage 3 | 7/10 (70.0) | 49/109 (45.0) |  |
| Serum creatinine, μmol/L, median (interquartile range) | 82.70  (68.78, 101.98) | 83.65  (64.05, 113.88) | 0.927 |
| eGFR, ml/min/1.73m^2^, mean ± SD | 70.80 ± 24.66 | 75.58 ± 29.98 | 0.594 |
| Stage of kidney function, N (%) | | | 0.393 |
| Stage 1&2 | 9/12 (75.0) | 82/124 (66.1) |  |
| Stage 3&4&5 | 3/12 (25.0) | 40/124 (33.9) |  |

Note: There were missing values in stage of hypertension (1 missing), history of hyperlipidemia (1 missing), history of hyperuricemia (1missing), history of hyperhomocystinemia (1 missing), stage of urine microalbumin (19 missing), stage of kidney function (2 missing), Data are displayed as means ± SD, N (%), or median (interquartile range) and compared by Student t-test, Mann-Whiteney U test or chi-square test where appropriate. * indicates p value <0.05.

IOP, Intraocular pressure; DR, Diabetic retinopathy; HDL, High-density lipoprotein cholesterol; LDL, Low-density lipoprotein cholesterol; HCY, Serum homocysteine; ACR, Urine albumin creatine ratio.
